# Differential impacts of pregnancy-induced hypertension and chronic hypertension on maternal and foetal health outcomes: a systematic review of outcomes and risk factors

**DOI:** 10.1007/s00404-026-08503-2

**Published:** 2026-06-22

**Authors:** Joseph Amihere Ackah, Wisdom Senanu Mensah, Xiangyan Chen, Ellen Dyer, Uchendu Comrade Chukwanugo, Christian Ven Emery, Theophilus N. Akudjedu

**Affiliations:** 1 Department of Health Technology and Informatics, The Hong Kong Polytechnic University, Kowloon, Hong Kong SAR, China; 2 Faculty of Health and Social Sciences, Institute of Medical Imaging and Visualisation, Bournemouth University, Bournemouth, UK; 3 Division of Science, Engineering and Health Studies, College of Professional and Continuing Education, The Hong Kong Polytechnic University, Kowloon, Hong Kong SAR, China; 4 Radiology Department-Ultrasound Unit, Cambridge University Hospitals NHS Foundation TRUST, Cambridge, UK; 5 Department of Radiology, Cross-Sectional Imaging Unit, University Hospital Southampton, Southampton, UK; 6 Faculty of Health, Education and Life Sciences, Birmingham City University, Birmingham, UK

**Keywords:** Chronic hypertension, Pregnancy, Foetal demise, Antenatal care, Pregnancy-induced hypertension, Pre-eclampsia, Obstetric ultrasound

## Abstract

**Introduction:**

Hypertensive disorders complicate approximately 5–10% of pregnancies globally, significantly impacting maternal and foetal health. To address the distinct impacts of chronic hypertension and pregnancy-induced hypertension (PIH) on foeto-maternal health, this review delineated their associated distinct risk factors and diagnostic markers, providing evidence-based insights to guide targeted, patient-centred management of hypertensive pregnancies.

**Methods:**

A systematic search of Web of Science, PubMed, and SCOPUS was conducted using predefined criteria. Observational studies were rigorously screened and quality assessed. Data were extracted and narratively synthesised, with an emphasis on maternal and foetal outcomes, diagnostic modalities, and risk modifiers.

**Results:**

The analysis confirmed that intrauterine foetal demise, intrauterine growth restriction, low birth weight, and neonatal as well as maternal mortality are major adverse outcomes of hypertensive pregnancies. The evidence demonstrates that whilst chronic hypertension and PIH confer considerable risk, PIH often results in more sudden and severe clinical deterioration, especially in the absence of vigilant prenatal monitoring and timely intervention. The findings underscored the value of targeted, patient-centred care: women with chronic hypertension benefit from early and sustained surveillance, whereas those with PIH require prompt escalation of monitoring following diagnosis, especially in the second trimester. Notably, comorbid systemic illnesses and advanced maternal age compound risks across hypertension categories, and diagnostic modalities, particularly ultrasound Doppler, are pivotal for early risk stratification and management.

**Conclusion:**

Effective management of hypertensive pregnancies requires early identification and individualised monitoring, recognising that chronic hypertension and pregnancy-induced hypertension differ in onset and progression, but both pose significant risks to maternal and foetal health.

**Supplementary Information:**

The online version contains supplementary material available at https://doi.org/10.1007/s00404-026-08503-2.

## Introduction

Pregnancy is a complex physiological journey characterised by profound biological changes that support foetal development and maternal adaptation. However, hypertensive disorders remain a significant obstetric challenge, ranking as the third leading cause of maternal morbidity and mortality globally [1] and a major contributor to intrauterine foetal demise and foetal growth restriction, with most adverse outcomes occurring in the late second and third trimesters [2, 3]. Hypertension adversely affects women across all reproductive ages and gestational stages, complicating approximately 5–10% of pregnancies worldwide and thereby constituting one of the most prevalent medical complications in pregnancy [4]. Of particular concern is the occurrence of foetal death in the mid-to-late gestational periods, which underscores the urgent need for a holistic understanding of hypertensive effects during these critical windows.

According to the American College of Obstetricians and Gynaecologists (ACOG), hypertension in pregnancy is classified into two primary subtypes: chronic hypertension, defined as blood pressure ≥ 140/90 mmHg diagnosed before 20 weeks’ gestation, and pregnancy-induced hypertension (PIH), which develops at or after 20 weeks’ gestation and includes gestational hypertension and pre-eclampsia [5–7]. Whilst both forms confer significant risk, emerging evidence suggests that the timing and nature of hypertensive onset differentially influence maternal and foetal outcomes, with the second trimester identified as a pivotal period for adverse sequelae [8–10].

Although prior research [11–13] has delineated the incidence, risk factors, and complications associated with hypertensive pregnancies, these studies often treat hypertensive disorders as a homogeneous entity, lacking a distinct comparative examination of chronic hypertension versus PIH. Moreover, the growing volume and heterogeneity of reproductive health literature pose challenges for clinicians and researchers striving to synthesise actionable knowledge [14–16]. This review addresses a critical gap by explicitly differentiating the impacts of pregnancy-induced and chronic hypertension on maternal and foetal health. Through a systematic synthesis of current evidence, this study aims to elucidate distinctive risk profiles, diagnostic markers, and clinical outcomes associated with each condition. By doing so, it seeks to enhance personalised, patient-centred management strategies and improve prognostic assessment in hypertensive pregnancies.

## Methodology and methods

### Protocol and registration

This study adopted a systematic review approach, following the updated version of the Preferred Reporting Items for Systematic Reviews and Meta-analyses (PRISMA) guidelines [17]. The eligibility criteria and search protocol were developed before the start of the study and registered with the International Prospective Register of Systematic Reviews (PROSPERO ID: CRD42023452372).

### Search strategy

Two reviewers (JAA and WSM) initially conducted a comprehensive independent literature search between July 2023 and August 2023. The search was updated on January 1, 2025 (CUC). The following databases (Web of Science, PubMed, and SCOPUS) were searched through the Bournemouth University Library.

For precise search parameters, review questions and search objectives were structured using the PICO framework (patient/population, intervention, comparison, and outcome) [18]. The search was conducted using the key words [(foetal demise or foetal death or stillbirth or IUFD) AND (hypertensive women or hypertensive disorders or pregnancy-induced hypertension or PIH or chronic hypertension or pregnant women) AND (second trimester or middle pregnancy or late trimester or late gestation or third trimester or 2nd trimester or 3rd trimester)] combined with Boolean operators (OR, AND) and truncation metrics (*). To avoid omitting relevant articles, the bibliographic lists of the selected studies were manually screened to include eligible articles. An expert librarian from Bournemouth University approved this search strategy for its effectiveness and robustness. A reference management programme (Mendeley) was used to manage the screening process and search results.

### Eligibility criteria

Following the PICOS framework, the eligibility criteria are as shown in Table 1**:**

**Table 1:** Inclusion criteria for studies on hypertension in pregnancy and foetal outcomes

Component	Details
Study design	Case–control and observational cohort studies (retrospective and prospective) published from 1990 to 2024 were included. This range was set to observe how the literature has transcended over the last two decades
Participants	Studies with participants who were pregnant women with hypertension (systolic pressure ≥ 140 mmHg and diastolic pressure ≥ 90 mmHg) as defined by established criteria [19]. There were no restrictions regarding age, sex, ethnicity/or disease risk groups. Studies reporting on foetal demise or pregnancy complications not induced by hypertension, or those of non-hypertensive women, and first-trimester complications were excluded. This was set up to ensure that the findings accurately represent the population under study
Interventions	Interventional and/or follow-up studies reporting obstetric and foetal medicine management amongst hypertensive patients were included
Comparators	Pregnant women living with no chronic hypertension or pregnancy-induced hypertensive disorders
Outcomes	Studies reporting maternal and foetal outcomes associated with hypertension-induced pregnancy or maternal chronic hypertension

Following the PICO framework criteria, inclusion was restricted solely to articles published in English or with English translations available. Meanwhile, the following types of content were excluded: review articles, letters to the editor, pictorial essays, postscripts, research letters, correspondence, unpublished data, opinion papers, commentaries, conference abstracts, theses and dissertations, and other topical proceedings. The criteria were set to ensure the inclusion of studies that had undergone a stringent peer-review process, thereby mitigating potential biases and the inclusion of opinion-based data.

### Data selection and screening

Following the initial search and removal of duplicates, two reviewers (JAA and WSM) independently screened the data for eligibility, utilising the web-based version of the Rayyan software [20]. An initial screening of titles and abstracts was performed, followed by detailed evaluations of full-text articles. When disparities or conflicts emerged during the screening and extraction phases, a third reviewer (INO) was involved to address and resolve them.

### Data extraction

Two members of the review team (JAA and WSM) independently conducted the data extraction, and an expert researcher (TNA) resolved disparities. We adopted a systematic approach to extract crucial details, including authorship, publication year, geographical location, sample size, participant characteristics, study design and duration, objectives, principal findings, and conclusions for each study (Supplementary Table S1).

### Quality assessment

The critical appraisal of the selected studies was conducted by two reviewers using the QATSDD tool [21], which comprises 16 criteria to facilitate a consistent, structured appraisal of the included randomised and non-randomised studies. Discrepancies were resolved by a third reviewer (ECV) in consultation with the research team. Each criterion was rated on a scale from 0 to 4 (0 = not at all, 1 = very slightly, 2 = moderately, 3 = complete). The overall quality score for each study was determined by summing the scores for these criteria. However, it is essential to note that these quality scores were not used as exclusion criteria. Studies were categorised based on their overall quality scores as follows: high quality (70% and above), moderate quality (50%–70%), or low quality (below 50%).

### Data synthesis and analysis

A result-based, convergent, and sequential synthesis design was employed to integrate multidisciplinary literature, consistent with established methodologies [22]. This approach enabled a comprehensive examination of the multifaceted relationship between maternal hypertension and foetal outcomes by synthesising evidence from varied study designs. Findings were synthesised through textual narrative and systematically presented in tabular form to enhance clarity and reproducibility. To address gaps identified in previous literature, this study synthesises and directly compares maternal and foetal outcomes associated with pregnancy-induced hypertension and chronic hypertension, providing novel insights into their differential risks and clinical implications. This method was particularly suited to managing the heterogeneity observed across outcome measures (see Supplementary Tables S1 and S2).

## Results

### Study characteristics

**Fig. 1:**
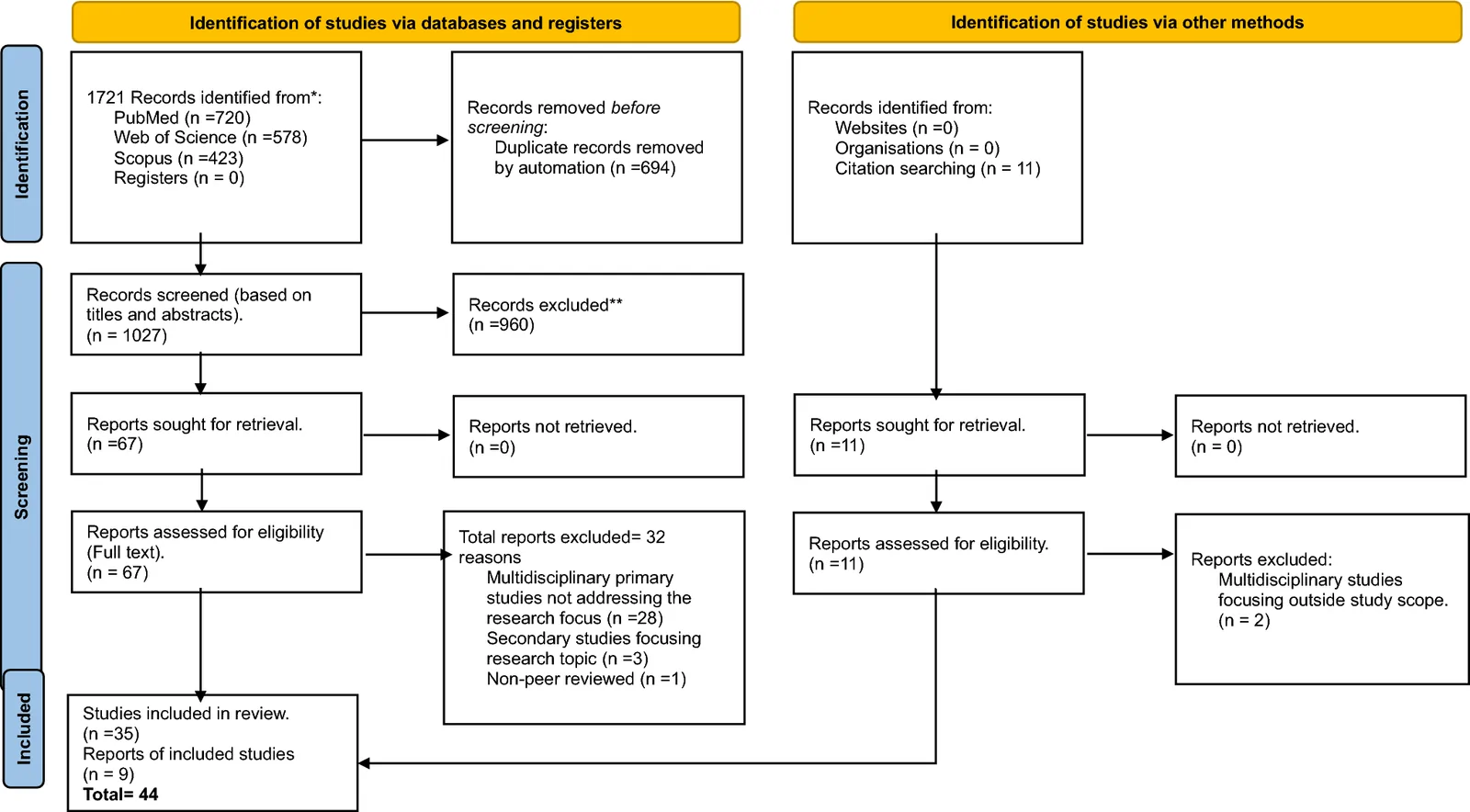
PRISMA flow chart

The included articles comprised 44 observational studies [8, 23–65] published from 1991 to 2025. Collectively, these studies included a pooled population of over 77 million pregnancies. From this population, 37,150 women with chronic hypertension and 730,135 with pregnancy-induced hypertension (PIH), encompassing gestational hypertension and pre-eclampsia, were identified and analysed across individual studies. A PRISMA flow diagram in Fig. 1 summarises the screening and selection process, including duplicates removal, eligibility assessment, and inclusion of additional references. From these data, 34 studies reported IUFD as a perinatal outcome [8, 23–25, 27, 29–33, 35–37, 39, 41–43, 45, 46, 48, 49, 52, 54–56, 58, 59, 61–65], whilst 23 studies reported intrauterine growth restriction (IUGR) as another perinatal outcome [17, 19, 21–24, 26, 27, 30, 33, 35, 36, 39, 40, 43, 44, 46, 48, 49, 54, 57, 59, 60]. Common postnatal outcomes include neonatal death, reported in 25 studies [8, 17, 19, 22, 23, 27, 31, 33, 35, 36, 39–41, 43–47, 51–53, 55–57, 60]; low birth weight, reported in 32 studies [8, 17, 19–23, 26–28, 30, 33, 36–40, 42, 43, 47–58, 60]; and maternal death, reported in 7 studies [41, 42, 55, 58, 62–64]. Notably, preterm birth and stillbirth were noted to be more frequent amongst hypertensive pregnancies, particularly where hypertension progressed rapidly during the second and third trimesters. Women with chronic hypertension were reported to exhibit a higher prevalence of baseline comorbidities, notably renal disease, systemic lupus erythematosus (SLE), obesity, and diabetes. Comorbidities of SLE and renal insufficiency were further associated with heightened risk for IUFD and IUGR, particularly when combined with hypertensive disorders. Additionally, advanced maternal age (≥ 35 years), obesity (BMI > 30), and the presence of chronic illnesses were strongly associated with poor maternal and foetal outcomes in both hypertension categories. Across studies, maternal deaths due to hypertensive complications, such as eclampsia, were reported in both women with underlying chronic hypertension as well as those with PIH in pregnancy. However, the risk is reported to be greater in PIH cases owing to acute onset and insufficient prenatal surveillance. Chronic hypertension-related complications were reported to manifest earlier in pregnancy, whilst PIH-related events arose distinctly after 20 weeks' gestation, with the second and third trimesters representing a critical period.

To enhance timely intervention and mitigate risks, ultrasonography plays an important role in the management of pregnancies complicated by hypertension, particularly in monitoring foetal growth and well-being and assessing placental function. For obstetric ultrasound monitoring and evaluation, umbilical and uterine arterial Doppler were the most effective parameters, as reported in 20 studies [17–23, 27, 30, 33, 34, 39, 40, 46, 48, 49, 54, 57–59] and 15 studies [18–23, 27, 30, 33, 40, 41, 55, 57–59], respectively. Table 2 summarises the distinct impacts of chronic hypertension and pregnancy-induced hypertension on maternal and foetal outcomes.

**Table 2:** Comparative maternal and foetal outcomes: chronic hypertension vs PIH

Domain	Chronic hypertension	Pregnancy-induced hypertension	Clinical implication
Sample size (included)	37,150 women (from 44 studies)	730,135 women (from 44 studies)	Robust data, substantial comparative pool
Maternal outcomes	- Greater association with baseline chronic comorbidities (renal disease, SLE, diabetes, obesity) - Higher prevalence of superimposed pre-eclampsia - Maternal death reported: 7 studies	- Acute onset and progression to severe hypertensive complications - More frequent development of pre-eclampsia/eclampsia - Maternal death reported: 7 studies	Early detection and management are critical
Foetal outcomes	- IUFD (intrauterine foetal demise) reported: 34 studies - IUGR (intrauterine growth restriction) reported: 23 studies - Low birth weight reported: 32 studies - Neonatal death: 25 studies - Preterm birth and stillbirth increased risk	- Comparable risk for IUFD/IUGR/Low birth weight - More rapid progression leads to acute foetal compromise - Neonatal death: 25 studies - Preterm birth and stillbirth increased risk	Surveillance intensity post-20 weeks is vital
Timing of adverse events	- Many complications arise pre-20 weeks or early in pregnancy due to a pre-existing condition	- Complications typically manifest after 20 weeks; the second and third trimesters most vulnerable	Critical window for PIH monitoring
Diagnostic/Monitoring modality	- Routine ultrasound and Doppler; early use - Umbilical/Uterine Doppler indices are frequently abnormal long before clinical symptoms - PI (pulsatility index) and RI (resistive index) thresholds used	- Ultrasound Doppler employed after 20 weeks - Acute changes in PI/RI are strongly associated with adverse outcomes - More intense serial Doppler monitoring required	Doppler indices can guide intervention
Risk modifiers/comorbidities	- Advanced maternal age (≥ 35), obesity (BMI > 30), SLE, chronic renal disease, diabetes	- Delay in diagnosis, lack of prenatal care, advanced age and obesity are also significant	Multifactorial, necessitating tailored care
Study quality (Risk of Bias)	- Moderate to high heterogeneity due to variability in study design/reporting - 16 high, 22 moderate, five low quality	- Same limitations; underreporting of methodology and setting - Quality remains a key issue	Strengthens the call for future robust studies

### Risk of bias and quality assessment outcomes

**Fig. 2:**
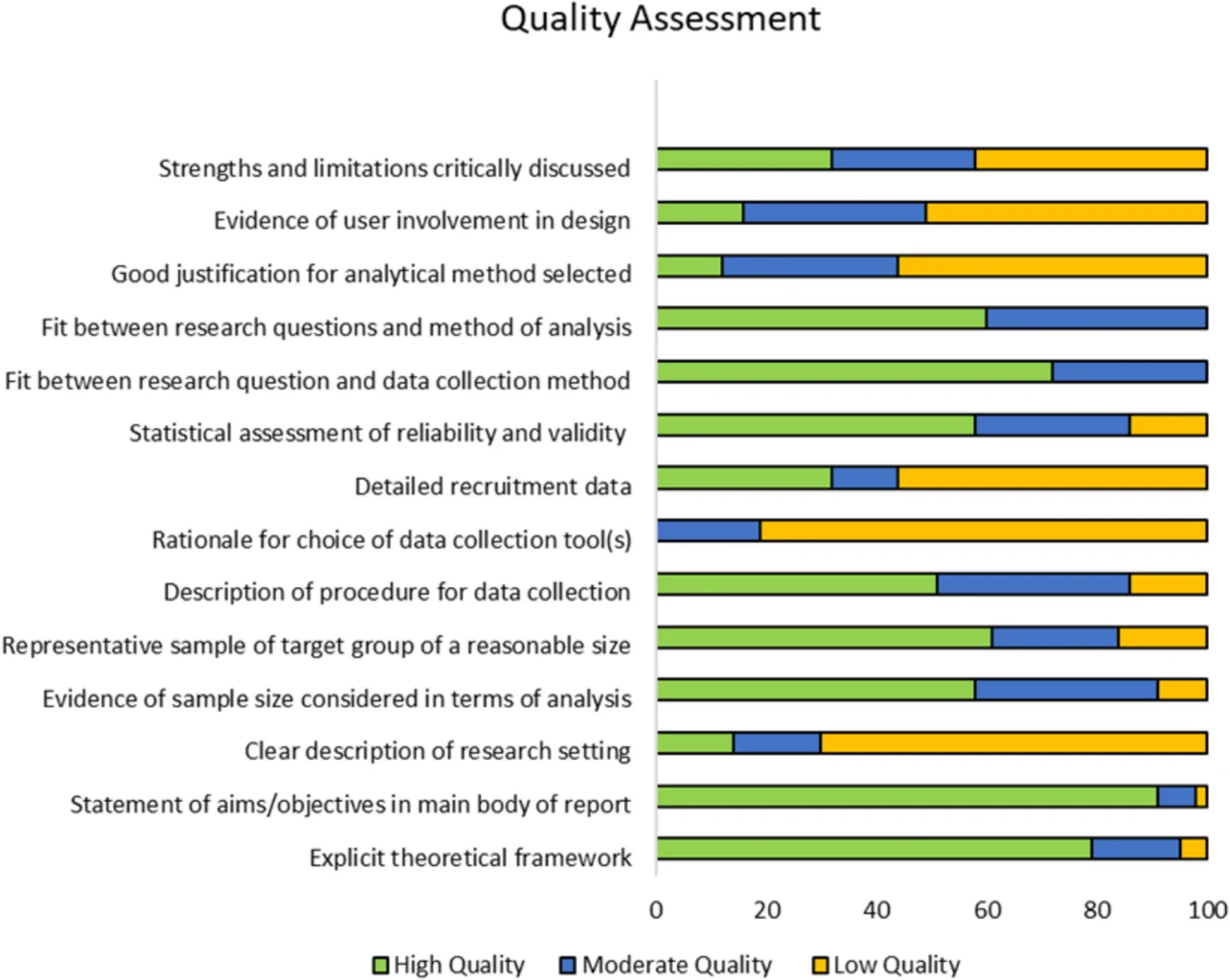
Quality assessment outcome

Figure 2 provides an overview of the quality assessment outcomes, highlighting 14 of 16 quantitative rating criteria, as all included studies were quantitative. The overall quality assessment of individual studies shows that 16 were high quality, 22 moderate, and 5 low quality (Fig. 2 and Supplementary Table S3). Whilst acknowledging the strengths in quality from Fig. 2 and Supplementary Table S3, the weaknesses highlighted here are intended to inform future researchers and clinicians of the need to exercise caution when drawing inferences from individual studies. It is also emphasised that researchers should adhere to the quality standards of the various quantitative research paradigms.

## Discussion

This systematic comparative review advances understanding of the differential impacts of chronic hypertension and PIH on maternal and foetal health. The synthesised evidence demonstrates that whilst both conditions confer considerable risk, PIH often results in more sudden and severe clinical deterioration, especially in the absence of vigilant prenatal monitoring and timely intervention. Notably, comorbid systemic illnesses and advanced maternal age compound risks across hypertension categories, and diagnostic modalities, particularly ultrasound Doppler, are pivotal for early risk stratification and management. Findings underscore the value of targeted, patient-centred care: women with chronic hypertension benefit from early and sustained surveillance, whereas those with PIH require prompt escalation of monitoring following diagnosis, especially in the second trimester. This study highlights clinical gaps, including delayed hypertension diagnosis and insufficient attention to risk modifiers, and identifies opportunities to improve outcomes through early detection and intervention. The key findings are detailed in subheadings, highlighting the diverse clinical outcomes and diagnostic examinations that inform evidence-based insights for effective decision-making and guide clinical efforts.

### Hypertension in pregnancy

The WHO estimated that there is at least one maternal death every seven minutes due to complications related to hypertensive disorders of pregnancy [66]. Hypertension in pregnancy is defined as systolic pressure ≥ 140 mmHg and/or diastolic pressure ≥ 90 mmHg [48, 67–69]. This review highlighted the risks associated with PIH and chronic hypertension. Unlike chronic hypertension, which is typically pre-existing and managed before pregnancy or before 20 weeks of gestation, PIH is shown to manifest suddenly during pregnancy, presenting unique challenges in its management. The acute onset and rapid progression of PIH has been associated with complications such as pre-eclampsia and eclampsia, which significantly impact foetal well-being and maternal health. Pre-eclampsia, characterised by blood pressure ≥ 140/90 mmHg after 20 weeks’ gestation, including cases progressing to severe pre-eclampsia defined by markedly elevated blood pressure (≥ 160/110 mmHg) with proteinuria and/or evidence of maternal organ dysfunction, is associated with serious complications for both the mother and the baby, including foetal demise, preterm birth, low birth weight, and stillbirth, as reported in other studies [66, 70]. Eclampsia, on the other hand, represents a severe complication in which seizures occur during pregnancy or the postpartum period, and may present with or without prior recognition of pre-eclampsia, posing an immediate risk to both maternal and foetal health [5]. This current study, therefore, strongly emphasises the need for clinicians and researchers to understand the differential impact of PIH and chronic hypertension on foetal and maternal outcomes. This understanding will aid in enhancing tailored management strategies and improving prenatal, perinatal, and postnatal care. Such efforts may include close monitoring of blood pressure, early detection of complications, especially in first-time pregnancies, and timely interventions to manage and stabilise maternal and foetal conditions, thereby improving pregnancy outcomes.

### Clinical considerations and potential mechanisms

This review highlights that most intrauterine foetal deaths in hypertensive pregnancies occur after the 28th week of gestation, a critical period marked by significant maternal haemodynamic changes. During pregnancy, systemic vascular resistance decreases due to vasodilatory hormone activity, reaching its lowest point around mid-gestation [71]. However, blood pressure naturally rises again in the third trimester as the cardiovascular system adapts to increased demands to support foetal growth. In hypertensive pregnancies, this adaptive process is often disrupted, contributing to increased risk of adverse outcomes [71]. Several key factors exacerbate the risk of materno-foetal complications during the second and third trimesters in women with hypertension. These include superimposed pre-eclampsia, maternal obesity (BMI > 30 kg/m^2^ as defined by ACOG), advanced maternal age (≥ 35 years), coexisting medical conditions, such as diabetes and renal disease, and delays in diagnosing and managing hypertensive disorders [59]. These factors interact to increase vulnerability to severe complications. The review observed a wide range of adverse foetal outcomes associated with hypertensive disorders, including preterm delivery (birth before 37 weeks), intrauterine growth restriction, low birth weight (< 2.5 kg), intrauterine foetal demise, and stillbirth [8, 17, 19, 22, 23, 27, 31, 33, 35, 36, 39–41, 43–47, 51–53, 55–57, 60]. On the maternal side, complications frequently reported were emergency caesarean sections, gestational hypertension progression, pre-eclampsia and eclampsia, gestational diabetes, respiratory distress, and both antepartum and postpartum haemorrhage [41, 42, 55, 58, 62–64]. These findings emphasise the importance of early detection, close monitoring, and timely intervention to mitigate risks and improve both maternal and foetal prognoses in hypertensive pregnancies.

### Maternal age as a critical factor

Maternal age has been identified as a significant factor influencing adverse outcomes for both mother and foetus [49]. Notably, women of advanced maternal age, generally defined as 35 years and older, exhibit a higher incidence of gestational hypertension, which emerges as a prominent risk factor in this demographical [63, 72]. Although the average maternal age at childbirth was reported as 30.9 years in 2022 by the Office for National Statistics (ONS), there is a global trend towards delayed childbearing [73]. Despite widespread recommendations advocating conception during early adulthood [25], no definitive age threshold has been universally established to predict the onset of hypertensive complications during pregnancy. This variability may be influenced by racial, geographical, cultural, and socio-economic factors, as well as individual lifestyle choices [74]. Research, including findings from Vanek and colleagues [49], suggests that many women are postponing childbirth as part of fulfilling broader “safety needs,” as conceptualised by Maslow’s hierarchy of needs [75, 76], which correlates with an increased prevalence of both chronic and pregnancy-induced hypertensive disorders. Consequently, the shift towards later maternal age may contribute to an increased burden of PIH and its associated maternal and foetal complications, including adverse perinatal outcomes in the mid-to-late trimesters.

### Hypertension with other comorbidities as critical factors

Pregnant women with hypertension who also present with additional comorbidities, such as systemic lupus erythematosus (SLE), underlying renal disease, gestational diabetes, cardiac conditions, and obesity, may face substantially elevated risks of foetal and neonatal mortality. These coexisting conditions create a multifaceted and interrelated risk profile that predisposes the foetus to adverse outcomes through complex pathophysiological mechanisms [4]. This review underscored that inadequate management of these comorbidities before and during early pregnancy significantly contributes to mid-gestation complications [49].

In particular, amongst women with SLE, disease activity, quantified using the Systemic Lupus Erythematosus Disease Activity Index (SLEDAI), is associated with an 8–32% risk of intrauterine growth restriction and pregnancy loss [23, 77]. This risk may be further increased when SLE coexists with hypertension. In addition, women with pre-existing SLE are at increased risk of developing hypertensive disorders during pregnancy and may experience disease flares, particularly during the second and third trimesters [31]. These flares are often precipitated by the discontinuation of chloroquine therapy, underscoring the importance of sustained disease management during pregnancy. Furthermore, renal disease, characterised by insufficiency and elevated creatinine, reportedly contributes to impaired placental function and increased risk of pre-eclampsia, preterm birth, and foetal growth restriction [78]. Gestational diabetes may also exacerbate vascular and metabolic stress, further raising the likelihood of adverse maternal and neonatal outcomes. Cardiac disease, on the other hand, is often associated with haemodynamic instability, increasing maternal morbidity and affecting foetal oxygenation and growth [79]. Obesity, a prevalent comorbidity, is independently associated with higher risks of pre-eclampsia, gestational diabetes, and caesarean delivery, compounding the challenges in hypertensive pregnancies [80].

### Timely diagnostics and challenges of hypertension in pregnancy

Delayed diagnosis of hypertension before or during pregnancy significantly exacerbates adverse maternal and foetal outcomes [28], a challenge that is particularly pronounced in low-resource settings [81]. This review underscores that late detection impedes timely clinical intervention and appropriate management, increasing the risk of severe complications such as foetal demise. Early and regular prenatal care, including systematic blood pressure monitoring, is essential for prompt identification and control of hypertensive disorders, thereby reducing potential harm to both mother and foetus. However, recent evidence indicates that many pregnant women delay seeking medical attention for signs and symptoms of gestational hypertension, contributing to postponed diagnosis and missed opportunities to mitigate risks [82]. Moreover, masked hypertension, characterised by regular blood pressure readings in clinical settings despite underlying hypertension, poses additional diagnostic challenges that further delay detection [82]. The importance of preconception screening for hypertension and associated comorbidities cannot be overstated, as early identification allows for close monitoring throughout pregnancy and improves outcomes for both mother and foetus [30, 66]. Accordingly, this review advocates holistic management strategies that prioritise the health of both mother and foetus, emphasising that interventions focussed solely on the foetus may inadvertently compromise maternal well-being and vice versa.

### Role of ultrasonography in hypertensive pregnancy diagnostics and management

Obstetric ultrasound, particularly Doppler imaging, is essential in managing hypertensive pregnancies by assessing maternal and foetal well-being. Widely used in high-resource settings, ultrasound accurately measures growth velocity, amniotic fluid volume, and placental function, enabling early detection of complications, such as intrauterine growth restriction and placental insufficiency [83, 84]. Doppler indices of uterine and umbilical arteries, combined with foetal biometry, assist clinicians in risk stratification and informed decision-making for pregnancies at risk of complications [67, 85].

### Standardised ultrasound findings from uterine artery Doppler

**Fig. 3:**
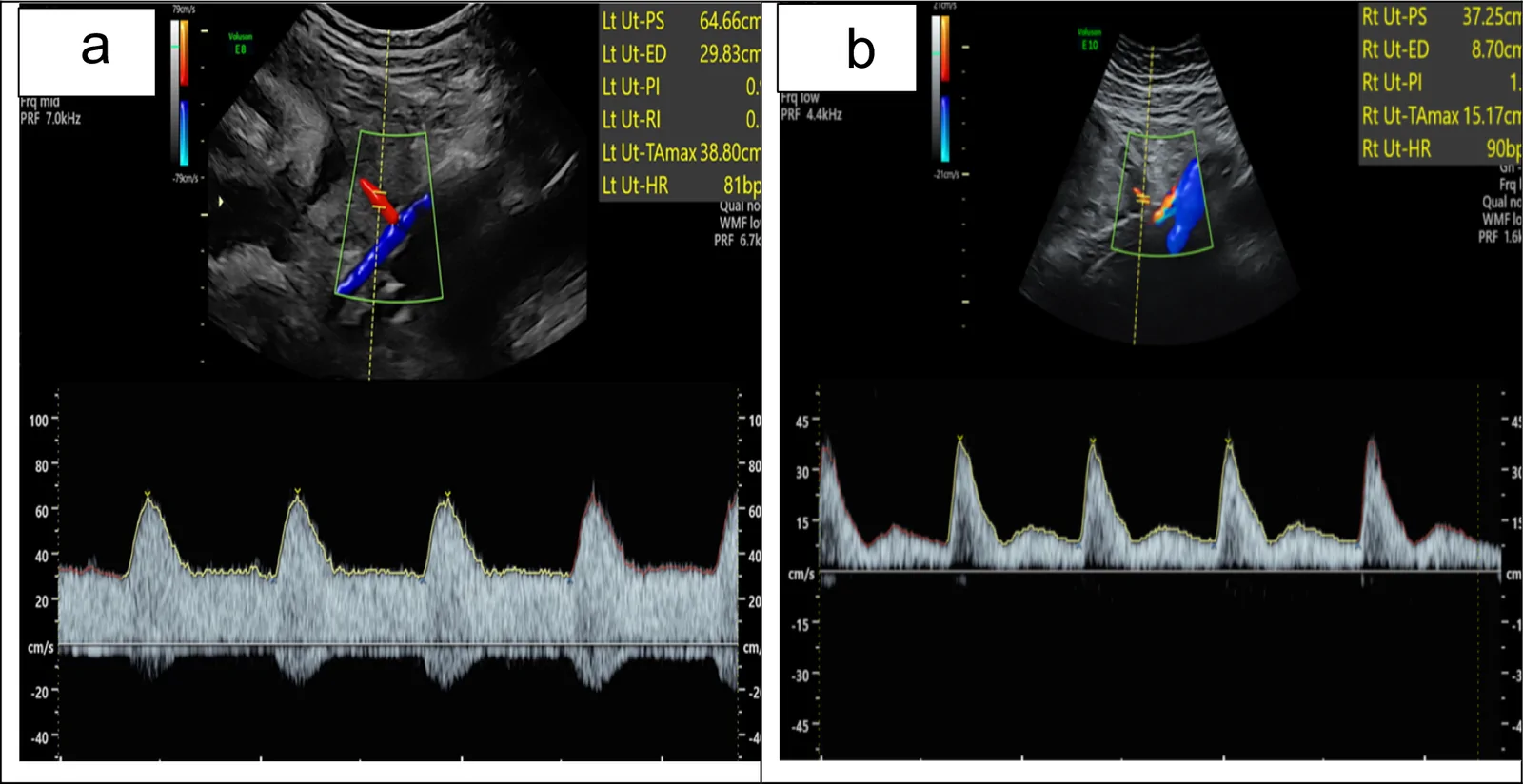
Illustration of Uterine Artery Ultrasonographic Doppler. **a** Is a normal uterine artery Doppler showing forward waveform and normal SD, RI, and PI values in a normotensive pregnancy. **b** Shows an abnormal uterine artery Doppler with a forward waveform, a high systolic peak, low diastolic blood flow velocities, and a post-systolic notch

Uterine artery Doppler is a vital diagnostic tool in obstetrics, providing key insights into maternal haemodynamic changes during pregnancy. Beyond detection, these measurements serve as significant predictors of adverse outcomes, including preterm delivery, antepartum haemorrhage, and pregnancy-induced hypertension (PIH). Whilst there is no universal consensus on the optimal Doppler index, the Society for Maternal-Foetal Medicine (SMFM) recommends the systolic/diastolic ratio, resistive index (RI), or pulsatility index (PI) [86], whereas the International Society of Ultrasound in Obstetrics and Gynaecology (ISUOG) favours the PI for its sensitivity [85]. The pulsatility and resistive indices are widely accepted markers of foetal well-being, with many centres, particularly in the UK, preferring PI due to its superior sensitivity in detecting uterine artery abnormalities [83]. Elevated mean PI and RI values have been consistently linked to adverse pregnancy outcomes, such as early pregnancy loss, second-trimester small for gestational age, and preterm birth [87]. Established normative thresholds at 20 to 24 weeks indicate average PI values of 1.09 (right artery) and 0.81 (left artery), with ranges between 0.53–1.58 and 0.58–1.83, respectively [71, 88, 89]. Detecting abnormal uterine artery Doppler waveforms enables clinicians to stratify risk early and tailor surveillance accordingly. This approach is exemplified by element 2 of the Saving Babies’ Lives Care Bundle (SBCLB) in NHS England [90], which underscores its clinical utility. Figure 3** (a, b)** illustrates normal and abnormal uterine artery Doppler waveforms.

### Standardised ultrasound findings from umbilical artery Doppler

**Fig. 4:**
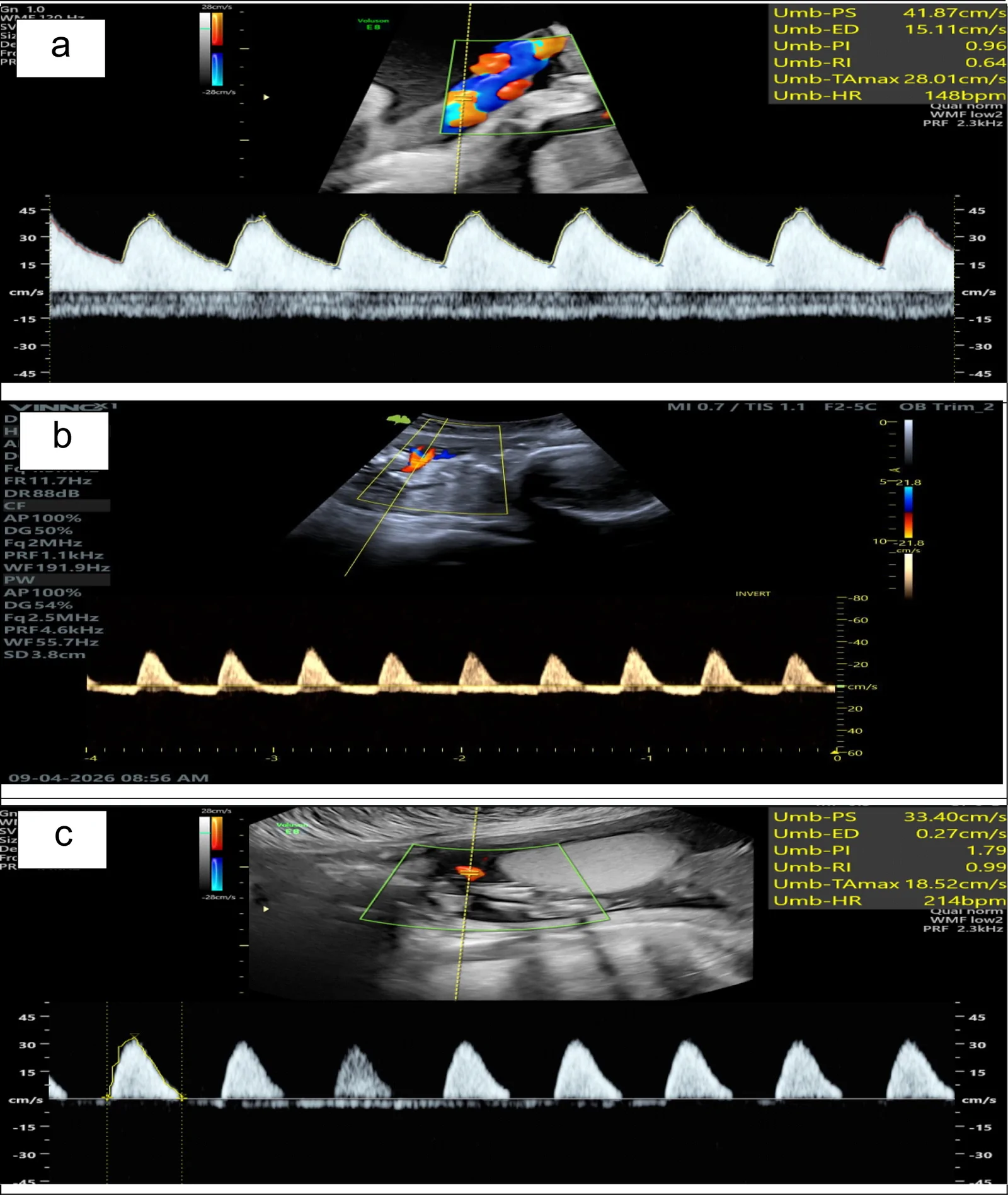
Illustration of Umbilical Artery Ultrasonographic Doppler. **a** Shows a normal umbilical artery Doppler with a forward waveform and normal SD, RI, and PI values in a normotensive pregnancy (29 weeks 2 days). **b** Depicts a typical waveform with reversed diastolic flow in a severe pre-eclampsia pregnancy (27 weeks 4 days). **c** Shows an absent end-diastolic flow in the umbilical artery in a foetus with early onset IUGR (28 weeks 5 days)

Abnormal umbilical artery Doppler indices in the second trimester reflect impaired cardiovascular adaptation [91]. This dysfunction is linked to poor placental perfusion, increasing risks of small for gestational age, preterm delivery, and intrauterine foetal demise [53]. Placental vascularisation in hypertensive pregnancies is consistently reduced during the second and third trimesters compared to normotensive pregnancies, regardless of treatment, further exacerbating adverse outcomes. Abnormal umbilical artery Doppler findings include elevated pulsatility index (PI) and, in severe cases, absent end-diastolic flow; reference charts aid interpretation across gestational ages [92, 93]. Prior to the development of the umbilical artery, early uterine artery Doppler screening in asymptomatic women is shown to be particularly valuable for identifying high-risk pregnancies, especially in those with chronic hypertension [65, 94]. However, some argue its use should be limited to symptomatic patients due to moderate sensitivity and specificity [32]. Our findings suggest that combining early uterine artery Doppler with biomarkers, such as Placental Growth Factor (PlGF), soluble fms-like tyrosine kinase 1 (sFlt-1), and maternal risk profiles, enhances the prediction of adverse pregnancy outcomes in asymptomatic women [14, 15]. Figures 4** (a–c)** illustrate normal and abnormal umbilical artery Doppler waveforms.

### Clinical implications from studies

In accordance with guidelines from the National Institute for Health and Care Excellence (NICE) [70], the WHO, and other leading regulatory bodies [16], the synthesised evidence from this review underscores that hypertensive disorders in pregnancy demand vigilant monitoring, emphasising both maternal and foetal health, given their dynamic and bidirectional influence on gestational progression across different pregnancy stages. Comprehensive foetal surveillance, including biometric measurements, such as femur length, head circumference, abdominal circumference, biparietal diameter, and amniotic fluid volume, is essential to assess foetal well-being and placental function. Serial ultrasounds at 28, 32, and 36 weeks, accompanied by umbilical artery Doppler studies, are recommended for women with chronic hypertension. In comparison, those with gestational hypertension should undergo ultrasounds every 2 to 4 weeks as clinically indicated. However, obstetric ultrasound alone may not fully capture the complexity of hypertensive pregnancy risks. Additional monitoring tools, such as cardiotocography and enhanced ultrasound protocols, are advised, particularly for pre-eclampsia cases with blood pressure ≥ 160/90 mmHg [95]. This review advocates the need for clinical studies exploring the effects of early interventions on the progression of maternal and neonatal outcomes across different maternal age groups. Future studies and clinical assessments must incorporate the established consensus thresholds for pregnancy examinations, as compiled in this review (Table 3), to ensure standardised evaluation and comparability across hypertensive pregnancy research and practice. Moreover, investigating the long-term impact of management strategies and the potential benefits of personalised treatment plans based on individual risk factors could lead to more effective, tailored care and improved health outcomes for mothers and infants.

**Table 3:** Normal Consensus Values from the Review

Parameter	Measurement		
Hypertension	≥ 140/90mmHg		
Pre-eclampsia	≥ 140/90 mmHg		
Proteinuria	Urine: protein 30mg/mmol		
	Albumin: creatinine 8mg/mmol		
Obesity	BMI > 30kg/m^2^		
Advanced maternal age	≥ 35years		
IUGR	< 2.5kg		
Preterm	Birth before 37 weeks		
Renal insufficiency	≥ 90mmol/litre		
Normal uterine artery Doppler indices range (20–24 weeks)	Right	Left	Average for right and left
PI	0.53–1.59	0.58–1.83	1.09 and 0.81
RI	0.59–0.65	0.37–1.16	0.59 and 0.65
SD	1.53–3.90	1.92–3.54	2.56 and 2.57
Normal umbilical artery Doppler Indices range (20–24 weeks)			
PI	0.765–0.585		
RI	1.265–0.829		
SD	4.068–0.829		

### Strengths and limitations

This review underscores the critical importance of timing in managing hypertension during pregnancy as delayed intervention may be as ineffective as no treatment at all. It also highlights the need to balance care between the mother and foetus as prioritising one over the other can have adverse consequences for both. Obstetric ultrasound remains the preferred modality for monitoring hypertensive pregnancies to optimise perinatal and postnatal outcomes. The clinical thresholds compiled here are validated and can be adapted according to national, regional, or international guidelines. However, relying solely on ultrasound may not fully capture all risk factors, as some essential clinical indicators could be overlooked without adjunctive assessments. A limitation of this review is the focus on English language studies, which may have overlooked relevant research published in other languages. However, non-English papers with available English translations were included to mitigate this limitation. Furthermore, whilst this review addresses management strategies for hypertensive disorders in pregnancy, detailed treatment protocols for specific complications and adverse outcomes fall outside its scope.

## Conclusion

This review comprehensively delineates the distinct impacts of chronic hypertension and pregnancy-induced hypertension on maternal and foetal health, emphasising their unique risk profiles and clinical trajectories. The findings highlight that whilst both conditions significantly elevate the risk of adverse outcomes, intrauterine foetal demise, growth restriction, and maternal complications, the timing, progression, and management strategies differ markedly. Early and accurate diagnosis, combined with comorbidity management, vigilant, individualised monitoring using ultrasonographic modalities and relevant biomarkers, is essential to mitigate risks. Thus, effective management of hypertensive pregnancies requires early identification and individualised monitoring, recognising that chronic hypertension and PIH differ in onset and progression. Still, both pose significant risks to maternal and foetal health.

### Statement of significance

#### Problem or Issue

Hypertensive disorders complicate up to 10% of pregnancies and are major contributors to maternal and perinatal morbidity and mortality. The impacts of pregnancy-induced hypertension and chronic hypertension on pregnancy outcomes remain insufficiently delineated.

#### What is already known?

Both PIH and chronic hypertension increase risks of intrauterine growth restriction, stillbirth, and adverse maternal outcomes. Existing studies often treat hypertensive disorders collectively, limiting precision in clinical guidance.

#### What does this paper add?

This review distinguishes the trajectories of PIH and chronic hypertension, demonstrating that PIH is associated with abrupt maternal and foetal health deterioration, whereas chronic hypertension necessitates sustained surveillance, reinforcing the need for condition-specific management.

## Supplementary Information

Below is the link to the electronic supplementary material.

## Data Availability

Data sharing does not apply to this study as no new data were created or analysed.

## Abbreviations

HTN: Hypertension
SLE: Systemic lupus erythematosus
FGR: Foetal growth restriction
CS: Caesarean section
APS: Antiphospholipid syndrome
ACL IgG: Anticardiolipin Immunoglobulin G
NLR: Neutrophil-to-lymphocyte ratio
SGA: Small for gestational age
NICU: Neonatal intensive care unit
CW Doppler: Continuous wave Doppler
PROM: Premature rupture of membranes
APO: Adverse pregnancy outcome
SLEDAI: Systemic lupus erythematosus disease activity index
PPIH: Pregnancy-induced hypertension
PPH: Postpartum haemorrhage
GA: Gestational age
MIFU: Mean intrauterine fluid
SCH: Subchorionic haemorrhage
AFP: Alpha-fetoprotein
PIH: Pregnancy-induced hypertension
HELLP: Haemolysis, Elevated Liver Enzymes, Low Platelet Count
LBW: Low birth weight
APGAR: Appearance, Pulse, Grimace, Activity, and Respiration (used for assessing the newborn’s condition)
