# Supplementary material for: Differential impacts of pregnancy-induced hypertension and chronic hypertension on maternal and foetal health outcomes: a systematic review of outcomes and risk factors
Source: Arch Gynecol Obstet. 2026 Jun 22;313(1):263. doi: 10.1007/s00404-026-08503-2 (PMC13538239; doi:10.1007/s00404-026-08503-2)
Supplement: Supplementary file 1 [file 404_2026_8503_MOESM1_ESM.pdf]

Supplementary Table

Table S1 *DATA EXTRACTION SHEET*

No	Study reference & Journal	Participants	Setting and design	Study Aim	Condition /Exposure under study	Fetal/Neonatal outcomes	Maternal outcomes	Key Findings and Conclusions	Quality Rating
1<br><br>(17)	(Aly et al., 2016)	Sample size= 91<br><br>No chronic HTN= 36<br><br>No Gestational HTN=	Clinical and laboratory findings of Pregnant women living with systemic lupus erythematosus in Egypt.<br><br>Prospective-observational study design	To evaluate the maternal and fetal outcomes of pregnancy in women with SLE and investigate the predictors and adverse pregnancy outcomes.	systemic lupus erythematosus	There was a significant fetal/neonatal adverse outcome from the study of which; 29 IUGR, 7 IUFD, 12 pre-terms and 3 neonatal deaths were recorded.<br>5 therapeutic abortions were recorded with 2 cases of severe hydrocephalus at	Systemic lupus erythematous flare was the dominant maternal adverse outcome; with renal flares (n=19) being the most frequent followed by serositis (n=3), pleurisy (n=2), and pericarditis (n=1). 12 out of the 91 women	The study revealed a significant association between hypertension and development of pre-eclampsia, and SLE flares.<br>Also, positive APS (antiphospholipid), Acl Igg (anticardiolipin immunoglobulin) is strongly associated with abortion and APS with fetal loss.	High quality

						15th week and 3 severe cases and lupus nephritis.	developed severe pre-eclampsia, 5 had premature rapture of membrane and 48 underwent caesarean surgery on account of previous C/S and cephalopelvic disproportion.		

2<br><br>(18)	(Aslan, Yeler, Yuvacı, et al., 2020)	Sample size= 175<br><br>No chronic HTN=<br><br>No Gestational HTN=	A retrospective study at Sakarya university education and research hospital- Turkey	To investigate the relationship between the neutrophil-to-lymphocyte ratio (NLR) and fetal outcomes in patients with severe pre-eclampsia	Neutrophil-to-Lymphocyte ratio in predicting fetal loss in pre-eclampsia	A total of 13 fetal losses, 77 preterm births and 66 low birth weight cases were recorded from the study.		In women with severe preeclampsia, a raised neutrophil-to-lymphocyte ratio in the third trimester, but not in earlier trimesters, was linked to foetal loss, predicting it with roughly 75 % sensitivity and 61 % specificity. Those who experienced foetal loss also frequently delivered preterm and had low-birth-weight infants.	Moderate quality
3<br><br>(19)	(Barton et al., 1997)	Sample size= 758	A hospital-based non-randomised	To compare the perinatal and maternal outcome	Hypertension with reference to age	Among 290 preterm births, hypertension was	Relatively low maternal complications	Hypertension is the leading cause of pre-term deliveries in both	Low quality

		No chronic HTN= 758<br><br>No Gestational HTN=	observational control study.	of mature and young women with pregnancy complicated by mild hypertension.		the leading indication, followed by ruptured membranes and spontaneous preterm labour across both younger and older mothers; there were 109 small-for-gestational-age infants, 156 NICU admissions, and five stillbirths.	were recorded, with five placenta abruption and 16 thrombocytopenia cases.	mature and young age groups.<br>Mature pregnant women pose a greater risk to perinatal outcome as compared to younger age groups. Thus, surveillance and adherence to nursing and physician instructions are recommended for hypertensive mothers, especially in older age groups.	

4<br><br>(20)	(Bernard et al., 2019)	Sample size= 6761<br><br>No chronic HTN=<br><br>No Gestational HTN= 202	A hospital based prospective observational study.	To investigate the association between the individual and combined effect of the exposure of antidepressants and anxiolytic drugs during the first and early second trimester and hypertensive disorders in pregnancy.	Antidepressants and anxiolytic exposures during early stages of pregnancy.		122 participants developed pre-eclampsia with evidence of increased risk of eclampsia.	The usage of antidepressants and anxiolytics before 16th week of pregnancy results in threefold higher risk of developing pre-eclampsia.	High quality

5<br><br>(21)	(Bewley et al., 1991)	Sample size= 925.<br><br>No chronic HTN=<br><br>No Gestational HTN=	Cross-sectional, observational study at the King's college hospital-London.	To determine the ability of a second-trimester CW Doppler examination of uteroplacental circulation to predict pre-eclampsia and SGA and to quantify its	Pre-eclampsia and IUGR in the second trimester.	12 cases of intrauterine fetal demise, 12 preterm births and 3 neonatal deaths were recorded.		There was heterogeneity in the outcome of the study; thus, the following submissions:<br>Not all antepartum haemorrhages are caused by placenta abruption<br>In early-onset pre-eclampsia, underlying renal disease may be underdiagnosed, adding	Moderate quality

				predictive properties.				to false-negative findings.<br>There may be false-positive and false-negative tests entirely due to biological variance.<br>It has been shown that the Doppler screening test is quick and reliable and with less than 1% failure.	

6<br><br>(22)	(Brew et al., 2022)	Sample size= 60,054.<br><br>No chronic HTN= 1,863<br><br>No Gestational HTN=	Retrospective observational study in 8 public Sydney hospitals- New South Wales.	To assess the impact of in-utero exposure to prolonged bushfires and pandemic restrictions on pregnancy and birth outcomes.	Bushfires and COVID-19 pandemic restrictions on pregnancy outcomes.	Stillbirth records of 474, low birth weight of 4,532, high birth weight of 5,430, preterm and small for gestational age (SGA) were recorded as adverse fetal outcomes.	The study recorded a significantly high rate of 8,972 unplanned C/S. Hypertension, gestational diabetes mellitus and pre-eclampsia were at a record of 1,863,	COVID-19 lockdowns led to more low-birth-weight infants—from first-trimester pollution and heat—and more unplanned C-sections due to delayed visits and reduced in-person care. Some babies weighed more, from foetal adaptation or	High quality

							10,747 and 1,102, respectively.	undiagnosed gestational diabetes.	

7<br><br>(23)	(Buyon et al., 2015)	Sample size= 385<br><br>No chronic HTN=<br><br>No Gestational HTN=	A prospective observational study at 8 USA and 1 Canadian site.	To identify predictors of adverse pregnancy outcomes in stable active systemic lupus erythematosus patients.	Systemic lupus erythematosus in pregnancy.	Fetal death was 18, 5 neonatal deaths, small for gestational age was 39. There were 29 PROM and 3 terminated pregnancies.	Gestational hypertension and preeclampsia were at a record of 33.	Overall, 19 % of pregnancies experienced adverse outcomes—foetal or neonatal death, preterm birth, or SGA, and these were predicted by maternal flare-ups, other disease activity, and rising C3 levels later in gestation. In those with added risk factors	Moderate quality

								(on antihypertensives with lupus anticoagulant positivity or of non-White/Hispanic ethnicity), the APO rate rose to 58 %, with a 22 % foetal/neonatal death rate.	
8 (24)	(Clemenza et al., 2022)	Sample size= 253<br>No chronic HTN= 4<br>No Gestational HTN= 8	A single center retrospective observational study at Careggi University Hospital; Florence-Italy	To evaluate a cohort of pregnant women with previous stillbirth and to assess the obstetrics outcome of the subsequent pregnancy by investigating the treatment of the underlying	Pregnancy outcome in women with history of stillbirth.	Fetal growth restriction (FGR) was 10, 1 stillbirth, 16 preterm deliveries and 22 miscarriages were recorded in this study.	28 patients suffered gestational diabetes, 5 with intrahepatic cholestasis and pulmonary tuberculosis of 25 had maternal adverse outcomes.	Foetal growth restriction, placental abruption, and other placental insufficiencies were the main causes of prior stillbirth. While subsequent pregnancies carry higher complication risks, multidisciplinary preconception evaluation and correction of modifiable factors led to	Moderate quality

				etiology using standard protocol.				high live-birth rates and no recurrent stillbirths.	
9<br>(25)	(Hernandez et al.,2002)	Sample size= 103<br>No chronic HTN= 8<br>No Gestational HTN= 5	A prospective observational study at Vall Hebron hospital – Barcelona; Spain.	To assess the outcome of pregnancy in systemic lupus erythematous patients and identify clinical and laboratory markers for fetal outcome and maternal flares	Systemic lupus erythematosis.	The study recorded several adverse outcomes aside from 68 live births: 15 spontaneous abortions, 12 stillbirths, 8 therapeutic abortions, 19 preterm, 24 intrauterine growth restrictions and 1 neonatal lupus flare.	34 maternal flares were recorded, 2 preeclampsia, 12 gestational proteinuria, 8 chronic hypertensive and 5 gestational hypertensive patients.	Women with antiphospholipid antibodies, active SLE, or hypertension require close monitoring. One-third of pregnancies experienced lupus flares, mostly in the second trimester or postpartum, especially if chloroquine was stopped, there were ≥3 pre-pregnancy flares, or SLEDAI was high. Foetal death, preterm birth, and IUGR were strongly linked to antiphospholipid	Moderate quality

								antibodies, low C3, and hypertension. Regular prenatal assessments—foetal echo, serial ultrasounds, and uteroplacental Doppler—aid early distress detection.	
10 (26)	Chiaie et al., 2001	Sample size= 30<br>No chronic HTN=<br>No Gestational HTN= 2	Prospective observational study at the Obstetrics and Gynaecology clinic of Parma university- Italy.	To establish whether asymptomatic normotensive pregnant women with an abnormal uterine Doppler velocimetry have haematological changes characteristic of congenital or acquired	Thrombophilic screening using Doppler velocimetry of uteroplacental vessels.	Intrauterine fetal demise, preterm birth.	Pre-eclampsia and C/S delivery.	Uterine-artery Doppler at 24–26 weeks can flag pregnancies at high risk for adverse outcomes, but its sensitivity and specificity are too low for routine screening in low-risk women. Thrombophilia-related blood abnormalities do not differ by Doppler result or outcome. In selected high-risk	Moderate quality

				thrombophilia, and if this information predicts pregnancy complications.				patients, its predictive accuracy rises to about 70 %, with a bilateral notch yielding a ~50 % PPV for pre-eclampsia and ~54 % for any adverse perinatal event.	
11 (27)	(Filippi et al., 2011)	Sample size= 240<br>No chronic HTN=28<br>No Gestational HTN= 17	A prospective observational study at the Fetal Medicine Unit, University College	To evaluate the use of second-trimester uterine artery Doppler to predict adverse pregnancy outcomes in	Relationship between fetoplacental protein and uterine artery Doppler findings.	A record of 42 small for gestational age, 19 low-birth weight, 10 preterm deliveries, 14 fetal loss, 11	There were 11 pre-eclampsia cases that resulted in emergency caesarean sections.	In women whose second trimester Down syndrome screening showed extreme fetoplacental protein levels, an abnormal uterine-artery Doppler	High quality

			hospital-London	women with extreme levels of fetoplacental proteins used for Down Syndrome screening.		spontaneous abortions and 3 stillbirth cases were noted from the study.		(notching or elevated pulsatility) was strongly linked to adverse outcomes, particularly growth restriction and low birth weight, while even unilateral notching carried heightened risk. Having multiple extreme markers further amplified this risk, and a normal Doppler result did not fully rule out complications. These findings support offering second-trimester uterine Doppler evaluation to guide management in this high-risk group.	

12<br><br>(28)	(Harrignton et al., 1997)	Sample size= 652<br><br>No chronic HTN= 30<br><br>No Gestational HTN=	Department of Obs & Gyn; St. George's Hospital- London, UK	To determine whether Doppler measurements obtained from the uterine and umbilical arteries in early pregnancy correlate with subsequent development adverse pregnancy outcomes.	Pre-eclampsia and IUGR prediction using uterine and umbilical artery Doppler.	The study reported 44 preterm births and 60 small for gestational age babies.	There were 40 recorded cases of maternal proteinuric pregnancy-induced hypertension and 23 antepartum haemorrhages.	There are differences in the umbilical and uterine artery Doppler indices in the first trimester among the cohort with adverse outcomes as compared to the cohort with normal delivery outcomes.<br>Abnormal Doppler values indicative of a failure to modify uterine circulation in early pregnancy are associated with adverse pregnancy outcomes: premature delivery, PPIH and SGA.	Moderate quality
13<br><br>(29)	Lees et al., 2001	Sample size= 5121<br><br>No chronic HTN=	A two-center prospective observational study at King's	To provide individualised risk prediction of severe adverse	Adverse pregnancy outcome prediction using	The study recorded 24 fetal deaths, 40 small for gestational age	22 cases of Preeclampsia were, however, the only adverse	The study identified ethnicity and smoking as key predictors of severe adverse pregnancy	Moderate quality

		No Gestational HTN= 2	College hospital, London and Queen Mary’s hospital Sidcup	pregnancy outcomes based on uterine artery Doppler screening.	uterine artery Doppler.	babies and 23 cases of placenta abruption.	maternal outcome.	outcomes, while maternal age and parity showed no significant effect. Smokers faced a notably higher risk than non-smokers. Additionally, uterine artery Doppler screening at 23 weeks was shown to enable individualized risk assessment for serious complications—such as foetal death, placental abruption, and pre-34-week delivery linked to preeclampsia and low birth weight.	

14<br><br>(30)	(Lesser et al., 2022)	Sample size= 32<br><br>No chronic HTN=<br><br>No Gestational HTN= 3	Obs&Gyn department of Sinai hospital-Baltimne	To evaluate maternal and fetal outcomes of low-risk singleton pregnancies that were diagnosed with Fetal Growth Restriction in mid to second trimester with initial fetal and maternal comorbidities.	Fetal Growth Restriction	The result from the study shows 8 NICU admissions, 5 neonatal deaths, 1 IUFD, 13 small for gestational age, 6 respiratory distress syndrome, 6 perinatal deaths, 17 preterm births and 2 bronchopulmonary diseases.	Maternal adverse outcomes were indifferent, such as 7 cases of preeclampsia, 13 unplanned caesarean sections. 3 gestational hypertensions were recorded.	Early onset FGR has a high association with SGA at delivery and adverse outcomes, hypertensive disorders of pregnancy, neonatal intensive care admission and neonatal deaths. Estimated fetal weight <5th% demonstrated a higher risk of adverse maternal and fetal outcomes compared with EFW of 5th -90th%.	High quality
15<br><br>(31)	(Mahmood et al., 2019)	Sample size= 622<br><br>No chronic HTN=<br><br>No Gestational HTN= 134	A Pakistan public hospital	To analyse the maternal and neonatal outcomes in women with third-trimester anaemia.	Iron Deficiency Anaemia	There were 138 low birth weight deliveries, 173 SGA, 93 pre-term deliveries, 18 stillbirths, 21 neonatal deaths	131 gestational hypertensions, 153 pre-eclampsia, 76 antepartum haemorrhage, 185 post-partum	Anaemic women with lower Hb values in the 1st and 2nd trimester had poor compliance with antenatal visits and a lower frequency of ion supplementation.	Moderate quality

						and 135 NICU admissions.	haemorrhage, prolonged obstructed labour of 115, urgent C/S of 105 and eclampsia of 5 were recorded as maternal outcomes in the anaemic group.	Anaemic women had nine times higher risk of PPH.<br>Maternal anaemia is associated with adverse maternal and neonatal outcomes. Focus should be placed on proper and adequate maternal nutrition. Women must be urged to seek antenatal care on regular basis.	
16<br><br>(32)	(McBride et al., 2015)	Sample size= 88,275<br><br>No chronic HTN= 21,896<br><br>No Gestational HTN=	Observational study at Vermont Oxford Network Center-North America	To evaluate the effect of maternal hypertension on mortality risk prior to delivery in infants 22-29weeks gestational age	Hypertension on mortality rate.	Small for gestational age perinatal death were the most significant fetal outcome		After adjusting for other risk factors, maternal hypertension remained an independent predictor of mortality across all gestational ages. Recognizing this protective effect can help	High quality

								clinicians counsel families and shape early-intervention decisions for incredibly low birth weight infants at the threshold of viability.	
17<br><br>(33)	(Monari et al., 2022)	Sample size= 783<br><br>No chronic HTN= 35<br><br>No Gestational HTN=61	A prospective observational study in Emilia Romagna-Italy	To evaluate the different modes of delivery in an Italian population of stillbirth, based on GA, parity, causes of death, obstetrics history and maternal characteristics	Mode of delivery in stillbirth cases	The study reviewed stillbirth cases within the population	Maternal outcomes in cases of stillbirth were multiple inductions in nulliparous women, higher rates of elective C/S, emergency and C/S, obesity, hypertension and diabetes.	Most stillbirths occurred after 28 weeks. Vaginal delivery was used in 84.3 percent, versus 15.7 percent by caesarean—of which 73.5 percent were emergency procedures. Although induction method did not vary by gestational age at stillbirth, women induced with prostaglandins were significantly more likely to achieve vaginal	High quality

								delivery than those receiving other methods.	
18 (34)	(Papastefanou et al., 2020)	Sample size= 124,443<br><br>No chronic HTN= 1569<br><br>No Gestational HTN=	A multicenter prospective observational study at King's College Hospital-London and Medway Maritime Hospital, Gillingham.	To develop a new model for predicting SGA neonates based on maternal history and characteristics.	Small for gestational age.		Chronic hypertension, diabetes mellitus and systemic lupus erythematosus were few maternal outcomes noted from the study.	Risk factors for having an SGA infant include shorter maternal stature and lower weight, non-White race, chronic hypertension, diabetes, lupus or antiphospholipid syndrome, IVF conception, smoking, prior preeclampsia or stillbirth, a history of delivering lower-weight or earlier-gestation babies, and a short interpregnancy interval. The study's new continuous-risk model—using these maternal	High quality

								demographics and medical history—accurately aligns predicted SGA risk with observed rates, making it a valuable tool for early screening.	
19<br><br>(35)	(Randrianaivo et al., 2006)	Sample size= 21,495<br><br>No chronic HTN= 4<br><br>No Gestational HTN=15	Prospective observational study in Reunion Island	To identify risk factors and causes of intrauterine fetal death in Reunion Island from 2001-2004	Intrauterine fetal death	Alongside intrauterine fetal deaths, congenital anomalies (n=104), perinatal infections (n=47), preterm delivery (n=122) and intrauterine fetal growth restrictions (n=41) were prominent fetal outcomes.	19 cases of hypertension, 12 antepartum haemorrhage, 8 gestational diabetes and 10 pre-eclampsia maternal outcomes were observed.	Between 2001 and 2004 in southern Reunion Island, there were 178 intrauterine foetal deaths among 21,495 births. Primiparity, maternal age ≥35, hypertensive disorders, and multiple gestations emerged as key obstetric risk factors. Infectious causes led the list (26%), followed by vascular growth restriction	Moderate quality

								(18%), cord and other specific perinatal conditions (14%), preeclampsia (10%), maternal health issues (8%), congenital anomalies (8%) and antepartum haemorrhage (7%). To lower MFIU rates, the authors recommend close surveillance of foetal growth alongside proactive management of diabetes, hypertension, and multifetal pregnancies.	
20 (36)	(Schutte et al., 2008)	Sample size= 27 No chronic HTN=	Retrospective observational study at the Dutch	To review the standard of care in cases due to hypertensive	Hypertensive-related maternal death.	Fetal death was a significant fetal outcome from the study.	Maternal death had a few causes; the highest being cerebrovascular	Between 2000 and 2004, 27 maternal deaths from hypertensive disorders were reported in the	Moderate quality

		No Gestational HTN=	Maternal Mortality Netherlands Society of Obs & Gynae.	disease-related maternal death.			accident (n=19). There were cases of multiorgan failure, acute respiratory distress syndrome, liver rupture and heart failure.	Netherlands, with substandard care identified in 96% of cases. Common deficiencies included inadequate diagnostic testing, mismanagement of hypertension and eclampsia, insufficient use of magnesium sulphate, poor patient stabilization, and delays in delivery, highlighting the need for enhanced education and training for both patients and healthcare providers.	
21 (37)	(Shen et al., 2017b)	Sample size= 1,054 No chronic HTN 11	A retrospective observational study at Shanghai	To observe and compare any differences between umbilical	Fetal development in hypertensive pregnancies.	There was a record of 6 spontaneous abortions, 8	There was a total of 394 caesarean section cases, of which 38 were	Gestational hypertension markedly elevates the risk of IUGR, preterm delivery, and perinatal	High quality

		No Gestational HTN 55	Sixth Hospital- Jiaotong University	and uterine blood flow detected by Doppler ultrasound in healthy and gestational hypertension pregnancies.		stillbirth cases, small for gestational age of 64 and 7 IUGR	among hypertensive women. Gestational hypertension and pre-eclampsia were also recorded.	complications, and is reflected in abnormal uterine and umbilical artery Doppler waveforms. In a cohort of over a thousand pregnancies, women were grouped into healthy, hypertensive, and high-risk hypertensive. Those in the high-risk hypertension group showed the greatest increases in uterine artery resistance and pulsatility indices during both mid- and late gestation. Overall, hypertensive pregnancies—especially	

								high-risk cases—were linked to higher preterm birth rates, more growth restriction, and smaller newborns compared to healthy pregnancies.	
22 (38)	(Singh et al., 2015)	Sample size= 12<br><br>No chronic HTN<br><br>No Gestational HTN=8	Prospective observational study at Maulana Azad Medical College-New Delhi, India	To evaluate the maternal and fetal outcomes in pregnancies with Takayasu aortoarteritis and whether early intervention of renal artery involvement is associated with improved outcomes.	Takayasu aortoarteritis	5 abortion cases, 1 preterm delivery,2 NICU admissions and 1 neonatal death were observed.	There were 8 gestational hypertension and 2 cases of preeclampsia	Women with renal artery involvement who do not receive intervention face significantly worse pregnancy outcomes—including gestational hypertension, pre-eclampsia, miscarriage, preterm birth, IUGR, foetal demise, and neonatal death—whereas preconception treatment, such as percutaneous transluminal balloon angioplasty, can	Moderate quality

								normalise blood flow and has been shown to enable successful pregnancies free of hypertension and growth restriction.	
23<br><br>(39)	(Trudell et al.; 2017)	Sample size= 64,173<br><br>No chronic HTN= 1,430<br><br>No Gestational HTN=	Midwestern United States quaternary referral center: a retrospective observational study.	To generate a clinical prediction tool for stillbirth that combines individual maternal risk factors to provide an evidence-based approach to the estimation of stillbirth risk.	Stillbirth prediction	A total of 464 stillbirth cases were recorded in the study. Anomalies and aneuploidy were at a record of 6,487	Maternal outcomes were pre-eclampsia (n=4,587), pre-gestational diabetes of 1,112 and gestational diabetes of 3,020.	The study devised a stillbirth-prediction tool for pregnancies beyond 32 weeks (excluding anomalies), which showed modest ability to distinguish risk. It generates a simple score to stratify women for targeted antenatal testing and, while it awaits independent validation, has the potential—if testing is begun at higher risk scores—to	High quality

								meaningfully lower late stillbirth rates and help achieve national reduction goals.	
24 (40)	(Gracia et al., 2004)	Sample size= 154<br><br>No chronic HTN<br><br>No Gestational HTN	A retrospective observational study at Complejo Hospitalario-Panama	To determine the perinatal outcome associated with severe chronic hypertension in pregnancy of more than 20 weeks of gestation	Chronic hypertension	There were adverse fetal outcomes such as: preterm rate of 92, fetal distress of 35, 12 stillbirth cases, 6 neonatal deaths, 13 respiratory distress syndrome, bronchopulmonary dysplasia of 8, FGR of 23 and pneumonia of 3 from the study.	110 maternal caesarean sections were carried out, and 120 preeclampsia were recorded.	Though outcomes in pregnancies complicated by subchorionic hematoma have improved compared to earlier reports, high rates of preterm birth, caesarean delivery, superimposed preeclampsia, placental abruption, and perinatal mortality persist. In this cohort, 78 % of women developed superimposed preeclampsia, delivering on average at 34.5 weeks with a mean birth weight	Moderate quality

								of 2,329 g; foetal growth restriction occurred in 18.5 % of cases.<br>Neonatal morbidity and mortality were driven chiefly by pulmonary complications and sepsis, and each additional week of gestation markedly boosted perinatal survival.	
25<br><br>(41)	(Waller et al., 1991)	Sample size= 612<br><br>No chronic HTN=<br><br>No Gestational HTN=	California AFP screening programme- a prospective observational study in California.	To investigate the possibility that serum alpha-fetoprotein level can be used to predict perinatal death at relatively late gestational age and provide	Alpha-fetoprotein levels in pregnancy	85 fetal deaths and 16 fetal anomalies were recorded from the study.		There was an increased maternal level of serum alpha-fetoprotein with an associated risk of fetal death.<br>The odds of fetal death were high if the mother were of black race, less than 20 years of age or	Low quality

				accurate risk estimates for various high levels of AFP.				more than 35 years of age.	
26 (42)	(Villar et al., 2006b)	Sample size= 39,615<br><br>No chronic HTN=<br><br>No Gestational HTN= 2,748	A multicenter cluster randomized study at: Rosario-Argentina, Havana-Cuba, Jeddah-Saudi Arabia and Khon Kaen-Thailand	To compare the determinants and perinatal outcomes associated with preeclampsia, gestational hypertension and unexplained intrauterine growth restriction (IUGR).	Pre-eclampsia, hypertension in pregnancy and IUGR.	There was a significant number of fetal deaths, neonatal deaths and early/late preterm deliveries, IUGR.	Maternal anemia, gestational hypertension, undernutrition, post-partum hemorrhage, and pre-eclampsia, amongst others, were maternal outcomes from the study.	Both first-time and experienced mothers can develop preeclampsia and IUGR, with preeclampsia carrying higher risks of later diabetes, renal and cardiac problems, UTIs, and chronic hypertension; intriguingly, smoking appears to slightly lower preeclampsia risk. Gestational hypertension, even without full preeclampsia, also	Moderate quality

								contributes to perinatal complications, underscoring the value of timely delivery when blood pressures rise.	
27<br><br>(43)	(Vanek et al., 2004)	Sample size= 114,965<br><br>No chronic HTN= 1807<br><br>No Gestational HTN=	A retrospective observational study at Soroka University Medical Center-Israel	To determine risk factors for the occurrence of chronic hypertension during pregnancy, and to investigate pregnancy outcome while controlling for superimposed preeclampsia.	Chronic hypertension in pregnancy	Intrauterine growth restriction (IUGR), perinatal mortality, small for gestational age, polyhydramnios, and placental abruption were the fetal outcomes observed in this study.	A significant number of the women from the study developed diabetes mellitus, had a blood transfusion, post-partum delivery. There was also an increase in Caesarean deliveries.	The study revealed a higher rate of perinatal mortality amongst pregnancies complicated by chronic hypertension. Chronic hypertension is not the sole risk factor for placenta abruption but rather a complication of superimposed preeclampsia.<br>Women of advanced age are more likely to develop chronic hypertension due to an increase in delayed	Moderate quality

								childbearing age amongst women over the past decade. The most common risk factor for developing chronic hypertension is diabetes mellitus, previous perinatal death, recurrent abortions, and women who have experienced infertility treatment.	
28 (44)	(Marchand et al., 2016)	Sample size= 413 No chronic HTN No Gestational HTN 14	A multicenter cross-sectional study at Ulaanbaatar, Selenge and Bulgan-Mongolia	To estimate the prevalence of preeclampsia in a contemporary population of Mongolian women in urban and rural areas.	preeclampsia	Small for gestational age and preterm were the most adverse fetal outcomes.	Upper edema, visual change and severe headaches were maternal adverse outcomes due to preeclampsia.	A higher proportion of women diagnosed with preeclampsia were from urban settlements. Maternal BMI has a positive association with preeclampsia diagnosed by a physician.	High quality

								In resource-limited settings, physicians diagnosed of preeclampsia can be overestimated, especially in urban areas. This overestimation may be beneficial, as it provides improved care for those at higher risk without resulting in adverse effects.	
29 (45)	(Ushida et al., 2021)	Sample size= 17,119<br><br>No chronic HTN<br><br>No Gestational HTN 698	A multicenter retrospective study at Aichi and Gifu – Japan.	To investigate the association between maternal low birth weight and subsequent risk for hypertensive	Low-birthweight and hypertensive disorders.	Low-birthweight and high-birthweight	Pre-eclampsia and hypertensive disorders in pregnancy.	From the study, maternal low birthweight (<2500g) is an independent risk factor for hypertensive disorder in pregnancy but not for preeclampsia.	Moderate quality

				disorders and preeclampsia.				The overlap between hypertensive disorders in pregnancy and foetal growth restriction should result in a higher risk of HDP in light-for-dates infants. Surprisingly, birthweight <2500g did not have HDP and preeclampsia, but rather birthweight >4000g had higher risks for these disorders.	
30<br><br>(46)	(Sibai et al., 2011)	Sample size= 739<br><br>No chronic HTN = 369<br><br>No Gestational HTN	A multisite observational study at Recife, Campinas, Botucatu, Porto Nagre-Brazil.	To compare the rates of superimposed pre-eclampsia and adverse outcomes in women with chronic hypertension with	Pre-eclampsia	The fetal outcomes from the study include a high rate of pre-term delivery, placenta abruption, small for gestational	Superimposed pre-eclampsia and pre-eclampsia were the major maternal outcomes.	17.8% of women with chronic hypertension developed superimposed pre-eclampsia.<br>There is no significant difference between women with or without pre-eclampsia that	High quality

				or without pre-eclampsia.		age, respiratory distress syndrome.		developed superimposed pre-eclampsia.<br>Maternal BMI, age or smoking status during pregnancy had no direct relation with the rate of superimposed pre-eclampsia but rather maternal systolic and diastolic pressure at enrolment.<br>There is a higher rate of preterm delivery <37weeks amongst women with prior pre-eclampsia.	
31<br><br>(47)	(XK. Chen et al., 2006)	Sample size= 17,432,987<br><br>No chronic HTN=	A large population-based retrospective study across	To assess the association between pregnancy-induced	Pregnancy-induced hypertension against neonatal death.	Neonatal death, pre-term birth and small for gestational age were the few	Pregnancy-induced hypertension, pre-eclampsia and eclampsia.	There was a comparatively lower neonatal death among preterm births from the PIH group than the non-	Moderate quality

		No Gestational HTN=676,392	50 states in the USA.	hypertension (PIH) and infant mortality.		neonatal outcomes from the study.		PIH group, but a higher neonatal death among PIH in term pregnancy as compared to the non-PIH group. This finding can be explained by the sum-up of pre-eclampsia, eclampsia, and gestational hypertension as PIH for the study. Also, most of the control preterm group from the non-PIH did not necessarily represent 'normal birth.' Thus, other high-risk conditions can be the cause of death in the non-PIH preterm birth.	

32 (48)	(Ahmad & Samuelsen, 2012)	Sample size= 2,121,371<br>No chronic HTN= 4,642<br><br>No Gestational HTN=32,068	A retrospective observational study from the medical birth registry of Norway.	To compare the proportion of offspring that were stillborn in pregnancies with preeclampsia, gestational hypertension and chronic hypertension.	Hypertensive disorders in pregnancy	Fetal death and preterm deaths were the most adverse fetal outcomes	Preeclampsia ad gestational hypertension	From 1967-2996; There was an increased risk of foetal death associated with hypertension as compared to the normotensive group. The Norwegian antenatal care programme and early detection improved the risk of foetal death from 1987-2006. However, there was an increase in preterm birth (<37weeks).	Moderate quality
33 (49)	(Adu-Bonsaffoh et al., 2017)	Sample size= 368<br><br>No chronic HTN 23<br><br>No Gestational HTN=184	A cross-sectional study at the Korle Bu Teaching Hospital in Accra- Ghana	To determine the perinatal outcomes of hypertensive disorders in pregnancy at a	Hypertensive disorders in pregnancy	NICU admission due to fetal asphyxia, stillbirth, neonatal death, IUGR and low birth weight.	Maternal death, preeclampsia and eclampsia, as well as an increased	The major perinatal outcomes were low birth rate (24.7%), preterm delivery (21.9%), respiratory distress (15.2%), and IUFD	High quality

				tertiary hospital in Ghana.			cesarean section rate.	(6.8%), with a perinatal mortality rate of 106/1000 births. Perinatal death was highest in the preeclampsia group and lowest in the chronic hypertensive group. Thirty percent of maternal deaths attributed to hypertensive disorders were due to suboptimal care.	
34 (50)	(Heard et al., 2004)	Sample size= 70,386<br><br>No chronic HTN=639	Observational study in South Australia.	To examine the effect of hypertensive disorders of pregnancy-on-pregnancy	Hypertension	Fetal death, NICU admission, Neonatal death and small for gestational age (SGA).	Increased rate of C/S, gestational hypertension, and preeclampsia.	The induction of labour and emergency caesarean sections were higher in all hypertensive groups compared to the normotensive group.	Low quality

		No Gestational HTN=5356		outcome in South Australia.				Superimposed preeclampsia has a higher perinatal and maternal morbidity than those with pregnancy-induced hypertension and chronic hypertension.<br>There was gross reduction in perinatal mortality for all hypertensive disorders especially for women with pre-existing hypertension.	
35<br>(51)	(Nurgaliyeva et al., 2020)	Sample size= 2548<br>No chronic HTN=<br>No Gestational HTN=	A retrospective cross-sectional study in 42 maternity facilities in Kazakhstani	To evaluate the prevalence of preeclampsia among Kazakhstani women and its	Preeclampsia	41 cases of bronchopulmonary dysplasia, 41 IUFD, 53 neonatal deaths and 118	Preeclampsia, arterial hypertension, chronic kidney disease, post-partum	There was an increased rate of placenta abruption among the non-preeclamptic group, even though preeclampsia is a risk	High quality

				associated maternal and neonatal outcomes.		perinatal deaths were recorded.	haemorrhage and placenta abruption were adverse maternal outcomes.	factor for placenta abruption. Early-onset preeclampsia is the most common type of preeclampsia, with bronchopulmonary dysplasia and early neonatal death as adverse outcomes. Primipara women experienced higher preeclampsia rates while multipara women had severe preeclampsia experiences.	
36 (52)	(Yücesoy et al., 2005)	Sample size= 255<br><br>No chronic HTN= 29	A retrospective cross-sectional study in Kocaeli	To determine the risk factors, prevalence, and maternal-perinatal outcomes in	Hypertensive disorders of pregnancy	Intrauterine Growth Restriction, oligohydramnios, NICU admission,	Placenta abruption, acute renal failure, disseminated intravascular	Placenta abruption, renal failure, and intravascular coagulation were the most common maternal adverse outcomes	Low quality

		N<u>o</u> Gestational HTN=	university-Turkey.	pregnant women with hypertensive disorders.		Intrauterine Fetal Demise.	coagulation, maternal mortality, HELLP syndrome, intracranial bleeding, retinal detachment and adult respiratory disease syndrome.	occurring in all hypertensive disorder groups: severe and mild preeclampsia and chronic hypertension. 5.7% of preeclamptic cases were diagnosed at the post-partum stage with 11.3% of them experiencing convulsions, hence the need for equal attention. Sociodemographic state is attributed to poor hypertensive disorders outcome as young women from low socio-demographic backgrounds, rural areas and primiparas	

								experienced eclampsia with convulsions.	
37<br><br>(53)	(Ananth & Basso, 2010)	Sample size= 57, 208,959<br><br>No chronic HTN=<br><br>No Gestational HTN=	A cross-sectional study in the USA	To investigate the relationship between pregnancy-induced hypertension and stillbirth and neonatal mortality in primipara and multipara women.	Pregnancy-induced hypertension.	Stillbirth and neonatal death were the most common fetal outcomes.	Pregnancy-induced hypertension.	There was no distinct classification of preeclampsia and non-proteinuric hypertension in this study. Pregnancy-induced hypertension occurred more in younger or older women, with a predilection for young Black primipara women. Pregnancy-induced hypertension was associated with increased risk of neonatal death amongst multiparous women.	Moderate quality
38	(Surányi et al., 2017)	Sample size= 226	Prospective observational	To investigate changes in	Hypertensive disorders of	Fetal hypoxia, apnea and IUGR.		In second and third trimester, placenta	Moderate quality

(54)		No chronic HTN= 43<br><br>No Gestational HTN=57	study at university of Szagel-Hungary	vascularization of the placenta and their effect on perinatal outcome Doppler indices of uterine arteries in second and third trimesters.	pregnancy in relation to placenta vascularization.			vascularization is significantly lower in hypertension compared to normotensive group despite antihypertensive treatment.<br>Chronic hypertension has similar placenta blood perfusion and adverse pregnancy outcome with normotensive women due to increased vascularization as a compensatory mechanism by the placenta.<br>IUGR with no proteinuria among gestational hypertension patients on	

								antihypertensive treatment was recorded. This could mean that maternal renal function is preserved, but placenta vascularisation is compromised in these patients.	
39<br><br>(55)	(Hauth et al., 2000)	Sample size= 4,302<br><br>No chronic HTN=<br><br>No Gestational HTN=1073	A multicenter observational study by the National Institute of Child Health and Human Development at New Mexico, Birmingham-Alabama, Cleveland-	To determine maternal and perinatal outcomes in nulliparous women with pregnancy-associated hypertension or pre-eclampsia.	Pregnancy outcome in hypertensive women undergoing a randomized calcium supplementation study.	Preterm, low-birth weight, fetal growth restriction, respiratory distress syndrome, intraventricular hemorrhage, NICU admission and fetal death.	Renal dysfunction, placenta abruption, induced labor, increased C/S rate and elevated liver enzymes.	Of 4,302 nulliparous women, 715 developed mild pregnancy-associated hypertension, 217 mild preeclampsia, thirty-seven severe pregnancy-associated hypertension and 109 severe preeclampsia. Foetal and neonatal deaths were common in the normotensive group than in the severe and	Low quality

			Ohio, Tennessee and Portland-Oregon.					mild hypertension and eclampsia groups. Women with severe preeclampsia were at the highest risk of adverse maternal and perinatal outcomes.	
40 (56)	(Roberts et al., 2005)	Sample size= 250,173<br><br>No chronic HTN= 1411<br><br>No Gestational HTN=10,864	Cross-sectional study in new South Wales	To determine current rates and outcomes of hypertensive disorders in pregnancy in a statewide population.	Hypertensive disease in pregnancy.	Fetal death, preterm delivery, small for gestational age, NICU admission, neonatal death.	High maternal death (10), with 6 directly related to hypertensive conditions, antepartum hemorrhage, post-partum hemorrhage, acute renal injury, and hepatic failure.	In this study, preeclampsia and gestational hypertension were combined in a single de-novo study, which is likely to mask the variation in outcomes.<br>Chronic hypertension in women has a high association with adverse foetus outcomes such as foetal death, NICU admission, whiles	Moderate quality

								women with gestational hypertension have a lower rate of morbidity and mortality. Chronic hypertension is prevalent among the elderly and nulliparous women.	
41 (57)	(Hinkosa et al., 2017)	Sample size=597<br>No chronic HTN= 4<br>No Gestational HTN=28	Retrospective unmatched case-control study in Nekemte Maternal hospital-Ethiopia	To determine both maternal and fetal risk factors and complications with hypertensive disorders in pregnancy.	Hypertension	Preterm, low birth weight, stillbirth and NICU admission.	Maternal death and abruptio placentae	Advanced maternal age is a significant risk factor for hypertensive disorders of pregnancy. Pregnant women from rural areas also demonstrated an increased rate of hypertensive disorder of pregnancy, which could be explained by the low level of education about	High quality

								the condition in these areas. Primigravida pregnancies had a higher risk of developing hypertensive disorders of pregnancy, due to the psychological and physical boredom they go through during pregnancy. Diabetes mellitus and familial history also have a strong association with hypertensive disorders in pregnancy.	
42 (8)	(Berhe et al., 2019)	Sample size= 798 No chronic HTN=	Prospective cohort study in 8 ANC hospitals in	To explore the effect of pregnancy induced	Pregnancy induced hypertension	Perinatal mortality, stillbirth, NICU admission, small for gestational	Preeclampsia, superimposed preeclampsia, gestational hypertension	Adverse perinatal outcomes (birth asphyxia, LBW, SGA, preterm birth, stillbirth) were higher in women	High quality

		N<u>o</u> Gestational HTN=20	Tigray state-Ethiopia	hypertension on perinatal outcome.		age, preterm delivery, birth asphyxia.		with pregnancy-induced hypertension compared to normotensive women. Poor access to healthcare and quality service is one of the major causes of poor perinatal outcome.<br>There was high incidences of adverse perinatal outcomes and pregnancy induced hypertension in Trigary. Improved perinatal outcomes by strengthening primary and secondary prevention, early diagnosis, and prompt management.	

43<br><br>(58)	(Seyom et al., 2015)	Sample size= 5415<br><br>No chronic HTN=<br><br>No Gestational HTN=121	Retrospective observational study in Mettu Karl referral hospital-Ethiopia	To highlight the management outcome and factors associated with pregnancy-related hypertension disorder in pregnancy	Pregnancy-related hypertension.	Low Birth Weight, Low APGAR score, stillbirth, early neonatal death, abortion, preterm birth	Renal failure, post-partum hemorrhage, placental abruption, thrombocytopenia and liver infarction.	Severe preeclampsia was the predominant hypertensive disorder, followed by eclampsia, mild preeclampsia, HELLP syndrome, gestational hypertension, and chronic hypertension. Most women (two-thirds) required both antihypertensive and anticonvulsant therapy, while about one-third received only antihypertensives. Spontaneous vaginal delivery was the norm, with caesarean delivery in under one-fifth of cases. Maternal and	Moderate quality

								foetal outcomes were closely tied to maternal age, socioeconomic status, and peak blood pressure.	
44 (54)	(Nie et al. 2024)	Sample size= 500<br><br>No chronic HTN 500<br><br>No Gestational HTN	Retrospective observational study in Beijing Chaoyang District Maternal and Child Health Care Hospital.	To explore the risk factors and maternal and foetal outcomes of preeclampsia amongst pregnant women with chronic hypertension.	preeclampsia	lower gestational age at delivery, lower birth weight, lower Apgar scores, increased umbilical artery resistance, abnormal amniotic fluid volume, and a higher incidence of fetal distress	HELLP syndrome, postpartum haemorrhage and placental abruption	The study examined preeclampsia in pregnant women with chronic hypertension, identifying three primary risk factors: a history of preeclampsia, hypertension lasting over three years, and the absence of systemic antihypertensive treatment. Preeclampsia was also associated with adverse outcomes, including increased incidences of maternal	

								HELLP syndrome. These findings emphasise the importance of early detection and intervention in high-risk pregnancies	
