# Supplementary material for: Differential impacts of pregnancy-induced hypertension and chronic hypertension on maternal and foetal health outcomes: a systematic review of outcomes and risk factors
Source: Arch Gynecol Obstet. 2026 Jun 22;313(1):263. doi: 10.1007/s00404-026-08503-2 (PMC13538239; doi:10.1007/s00404-026-08503-2)
Supplement: Supplementary file 2 [file 404_2026_8503_MOESM2_ESM.pdf]

Supplementary Table

Table S2. SYNTHESISED DATA

No	Study reference & Journal	Participants	Perinatal/Postnatal Outcomes					Ultrasound/Imaging Examinations		
			IUFD	IUGR	LBW	ND	MD	Uterine artery Doppler	Umbilical Artery Doppler	General Obstetrics
1<br>(17)	(Aly et al., 2016)	Sample size= 91<br>No chronic HTN= 36<br>No Gestational HTN= nr	√	√	√	√	x	x	√	√
2<br>(18)	(Aslan, Yeler, Yuvacı, et al., 2020)	Sample size= 175<br>No chronic HTN= nr<br>No Gestational HTN= nr	√	x	√	x	x	x	x	√
3<br>(19)	(Barton et al., 1997)	Sample size= 758<br>No chronic HTN= 758<br>No Gestational HTN= nr	√	√	√	√	x	x	√	√
4<br>(20)	(Bernard et al., 2019)	Sample size= 6761<br>No chronic HTN= nr<br>No Gestational HTN= 202	x	x	x	x	x	x	x	√
5	(Bewley et al., 1991)	Sample size= 925.<br>No chronic HTN= nr	√	x	√	√	x	√	x	√

(21)		No Gestational HTN= nr								
6<br><br><br>(22)	(Brew et al., 2022)	Sample size= 60,054.<br>No chronic HTN= 1,863<br>No Gestational HTN= nr	x	x	√	√	x	x	x	x
7<br><br><br>(23)	(Buyon et al., 2015)	Sample size= 385<br>No chronic HTN= nr<br>No Gestational HTN= nr	√	√	√	√	x	√	√	√
8<br><br><br>(24)	(Clemenza et al., 2022)	Sample size= 253<br>No chronic HTN= 4<br>No Gestational HTN= 8	√	√	√	x	x	√	√	√
9<br><br><br>(25)	(Hernandez et al.,2002)	Sample size= 103<br>No chronic HTN= 8<br>No Gestational HTN= 5	√	√	x	x	x	√	√	√
10<br><br><br>(26)	Chiaie et al., 2001	Sample size= 30<br>No chronic HTN= nr<br>No Gestational HTN= 2	√	x	x	x	x	√	√	√
11<br><br><br>(27)	(Filippi et al., 2011)	Sample size= 240<br>No chronic HTN=28<br>No Gestational HTN= 17	√	√	√	√	x	√	√	√

12<br><br>(28)	(Harrignton et al., 1997)	Sample size= 652<br>No chronic HTN= 30<br>No Gestational HTN= nr	x	x	√	x	x	√	√	√
13<br><br>(29)	Lees et al., 2001	Sample size= 5121<br>No chronic HTN= nr<br>No Gestational HTN= 2	√	√	√	x	x	√	√	√
14<br><br>(30)	(Lesser et al., 2022)	Sample size= 32<br>No chronic HTN= nr<br>No Gestational HTN= 3	√	√	√	√	x	√	√	√
15<br><br>(31)	(Mahmood et al., 2019)	Sample size= 622<br>No chronic HTN= nr<br>No Gestational HTN= 134	√	√	√	√	x	√	√	√
16<br><br>(32)	(McBride et al., 2015)	Sample size= 88,275<br>No chronic HTN= 21,896<br>No Gestational HTN= nr	x	√	√	x	x	x	x	x
17<br><br>(33)	(Monari et al., 2022)	Sample size= 783<br>No chronic HTN= 35<br>No Gestational HTN=61	√	x	x	x	x	x	x	√
18<br><br>(34)	(Papastefanou et al., 2020)	Sample size= 124,443<br>No chronic HTN= 1569<br>No Gestational HTN= nr	x	√	√	x	x	x	x	x

19<br><br>(35)	(Randrianaivo et al., 2006)	Sample size= 21,495<br>No chronic HTN= 4<br>No Gestational HTN=15	√	√	√	√	√	√	√	√
20<br><br>(36)	(Schutte et al., 2008)	Sample size= 27<br>No chronic HTN= nr<br>No Gestational HTN= nr	√	x	x	x	√	x	x	√
21<br><br>(37)	(Shen et al., 2017b)	Sample size= 1,054<br>No chronic HTN= 11<br>No Gestational HTN= 55	√	√	√	x	x	√	√	√
22<br><br>(38)	(Singh et al., 2015)	Sample size= 12<br>No chronic HTN= nr<br>No Gestational HTN=8	x	x	x	√	x	x	x	x
23<br><br>(39)	(Trudell et al.; 2017)	Sample size= 64,173<br>No chronic HTN= 1,430<br>No Gestational HTN= nr	√	x	x	x	x	x	x	√
24<br><br>(40)	(Gracia et al., 2004)	Sample size= 154<br>No chronic HTN= nr<br>No Gestational HTN= nr	√	√	√	√	x	√	√	√
25<br><br>(41)	(Waller et al., 1991)	Sample size= 612<br>No chronic HTN= nr<br>No Gestational HTN= nr	√	x	x	x	x	x	√	√

26<br><br>(42)	(Villar et al., 2006b)	Sample size= 39,615<br>No chronic HTN= nr<br>No Gestational HTN= 2,748	√	√	x	√	x	x	x	√
27<br><br>(43)	(Vanek et al., 2004)	Sample size= 114,965<br>No chronic HTN= 1807<br>No Gestational HTN= nr	√	√	√	√	x	x	x	x
28<br><br>(44)	(Marchand et al., 2016)	Sample size= 413<br>No chronic HTN= nr<br>No Gestational HTN= 14	x	x	√	x	x	x	x	x
29<br><br>(45)	(Ushida et al., 2021)	Sample size= 17,119<br>No chronic HTN= nr<br>No Gestational HTN= 698	x	x	√	x	x	x	x	x
30<br><br>(46)	(Sibai et al., 2011)	Sample size= 739<br>No chronic HTN= 369<br>No Gestational HTN=	√	√	√	√	x	√	√	√
31<br><br>(47)	(Xk et al., 2006)	Sample size= 17,432,987<br>No chronic HTN= nr<br>No Gest. HTN=676,392	x	x	x	√	x	√	x	√
32<br><br>(48)	(Ahmad & Samuelsen, 2012)	Sample size= 2,121,371<br>No chronic HTN= 4,642<br>No Gest. HTN=32,068	√	x	√	x	x	x	x	x

33<br><br>(49)	(Adu-Bonsaffoh et al., 2017)	Sample size= 368<br>No chronic HTN= 23<br>No Gestational HTN=184	√	√	√	√	√	x	x	√
34<br><br>(50)	(Heard et al., 2004)	Sample size= 70,386<br>No chronic HTN=639<br>No Gestational HTN=5356	√	√	x	√	x	x	x	x
35<br><br>(51)	(Nurgaliyeva et al., 2020)	Sample size= 2548<br>No chronic HTN= nr<br>No Gestational HTN= nr	√	x	x	√	x	x	x	√
36<br><br>(52)	(Yücesoy et al., 2005)	Sample size= 255<br>No chronic HTN= 29<br>No Gestational HTN=nr	√	√	x	√	√	x	√	√
37<br><br>(53)	(Ananth & Basso, 2010)	Sample size= 57, 208,959<br>No chronic HTN= nr<br>No Gestational HTN=nr	√	x	√	√	x	x	x	x
38<br><br>(54)	(Surányi et al., 2017)	Sample size= 226<br>No chronic HTN= 43<br>No Gestational HTN=57	x	√	√	x	x	√	√	√
39<br><br>(55)	(Hauth et al., 2000)	Sample size= 4,302<br>No chronic HTN= nr<br>No Gestational HTN=1073	√	√	√	x	x	x	√	x

40<br><br>(56)	(Roberts et al., 2005)	Sample size= 250,173<br>No chronic HTN= 1411<br>No Gest. HTN=10,864	√	x	√	√	√	x	x	x
41<br><br>(57)	(Hinkosa et al., 2017)	Sample size=597<br>No chronic HTN= 4<br>No Gestational HTN=28	√	x	√	√	√	x	x	x
42<br><br>(8)	(Berhe et al., 2019)	Sample size= 798<br>No chronic HTN= nr<br>No Gestational HTN=20	√	x	√	√	x	x	x	x
43<br><br>(58)	(Seyom et al., 2015)	Sample size= 5415<br>No chronic HTN= nr<br>No Gestational HTN=121	√	x	√	√	√	x	x	X
44<br><br>(59)	(Nie et al., 2024)	Sample size= 500<br>No chronic HTN 500<br>No Gestational HTN	√	√	√	x	x	x	√	√

Intrauterine fetal demise (IUFD), intrauterine growth restriction (IUGR), low birth weight (LBW), neonatal death (ND), maternal death (MD), not reported (nr)
