# Supplementary material for: Differential impacts of pregnancy-induced hypertension and chronic hypertension on maternal and foetal health outcomes: a systematic review of outcomes and risk factors
Source: Arch Gynecol Obstet. 2026 Jun 22;313(1):263. doi: 10.1007/s00404-026-08503-2 (PMC13538239; doi:10.1007/s00404-026-08503-2)
Supplement: Supplementary file 3 [file 404_2026_8503_MOESM3_ESM.pdf]

Supplementary table

TABLE S3. QATSDD RISK OF BIAS ASSESSMENT

	**Risk of Bias Rating**		
	**Low RoB (%)**	**Moderate RoB (%)**	**High RoB (%)**
1. Explicit theoretical framework	34 (79)	7 (16)	2 (5)
2. Statement of aims/objectives in main body of report	39 (91)	3 (7)	1 (2)
3. Clear description of research setting	6 (14)	7 (16)	30 (70)
4. Evidence of sample size considered in terms of analysis	25(58)	14(33)	4(9)
5. Representative sample of target group of a reasonable size	26(61)	10(23)	7(16)
6. Description of procedure for data collection.	22(51)	15(35)	6(14)
7. Rationale for choice of data collection tool(s)	0(0)	8(19)	35(81)
8. Detailed recruitment data	14(32)	5(12)	24(56)
9. Statistical assessment of reliability and validity of measurement tool(s) (Quantitative only)	25(58)	12(28)	6(14)
10. Fit between stated research question and method of data collection (Quantitative)	31(72)	12(28)	0(0)
11. Fit between stated research question and format and content of data collection tool e.g., interview schedule (Qualitative)	N/A	N/A	N/A
12. Fit between research questions and method of analysis	26(60)	17(40)	0(0)
13. Good justification for analytical method selected	5(12)	14(32)	24(56)
14. Assessment of reliability of analytical process (Qualitative only)	N/A	N/A	N/A
15. Evidence of user involvement in design	7(16)	14(33)	22(51)
16. Strengths and limitations critically discussed	14(32)	11(26)	18(42)

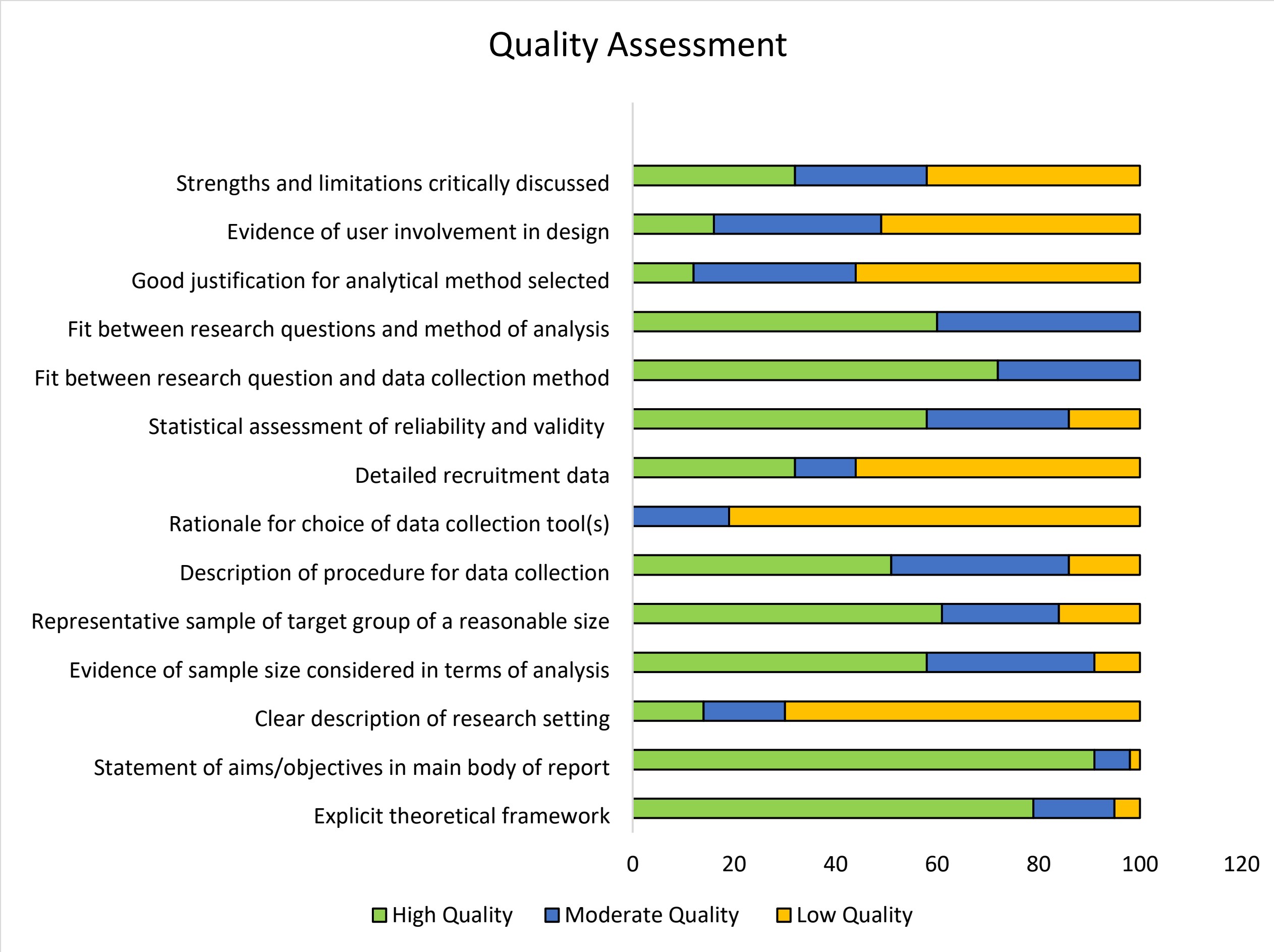

Quality Assessment
Strengths and limitations critically discussed
Evidence of user involvement in design
Good justification for analytical method selected
Fit between research questions and method of analysis
Fit between research question and data collection method
Statistical assessment of reliability and validity
Detailed recruitment data
Rationale for choice of data collection tool(s)
Description of procedure for data collection
Representative sample of target group of a reasonable size
Evidence of sample size considered in terms of analysis
Clear description of research setting
Statement of aims/objectives in main body of report
Explicit theoretical framework
0
20
40
60
80
100
120
High Quality
Moderate Quality
Low Quality
